# Chikungunya virus entry and infectivity is primarily facilitated through cell line dependent attachment factors in mammalian and mosquito cells

**DOI:** 10.3389/fcell.2023.1085913

**Published:** 2023-01-20

**Authors:** Judith Mary Reyes Ballista, Kerri L. Miazgowicz, Marissa D. Acciani, Ariana R. Jimenez, Ryan S. Belloli, Katherine E. Havranek, Melinda A. Brindley

**Affiliations:** ^1^ Department of Infectious Diseases, College of Veterinary Medicine, University of Georgia, Athens, GA, United States; ^2^ Department of Population Health, College of Veterinary Medicine, University of Georgia, Athens, GA, United States

**Keywords:** chikungunya, virus entry, attachment factors, receptors, arbovirus

## Abstract

Chikungunya virus (CHIKV) is the causative agent of the human disease chikungunya fever, characterized by debilitating acute and chronic arthralgia. No licensed vaccines or antivirals are currently available for CHIKV. Therefore, the prevention of attachment of viral particles to host cells is a potential intervention strategy. As an arbovirus, CHIKV infects a wide variety of cells in both its mammalian and mosquito host. This broad cell tropism might stem from CHIKV’s ability to bind to a variety of entry factors in the host cell including phosphatidylserine receptors (PSRs), glycosaminoglycans (GAGs), and the proteinaceous receptor Mxra8, among others. In this study, we aimed to determine the relevance of each attachment factor during CHIKV entry into a panel of mammalian and mosquito cells. Our data suggest that the importance of particular binding factors during CHIKV infection is highly cell line dependent. Entry into mammalian Vero cells was mediated through attachment to PSRs, mainly T-cell immunoglobulin mucin domain-1 (TIM-1). Conversely, CHIKV infection into HAP1 and NIH3T3 was predominantly mediated by heparan sulfate (HS) and Mxra8, respectively. Entry into mosquito cells was independent of PSRs, HS, and Mxra8. Although entry into mosquito cells remains unclear, our data denotes the importance of careful evaluation of reagents used to identify receptor use in invertebrate cells. While PSRs, GAGs, and Mxra8 all enhance entry in a cell line dependent manner, none of these factors are necessary for CHIKV entry, suggesting additional host factors are involved.

## 1 Introduction

Chikungunya virus (CHIKV) is a mosquito-borne alphavirus that can cause debilitating arthralgia and joint pain. Outbreaks of CHIKV were originally limited to Africa or Asia (Powers et al., 2000; Volk et al., 2010), but recent emergence introduced it throughout the Americas and Europe (Angelini et al., 2007; Rezza et al., 2007; Vega-Rua et al., 2013; Cassadou et al., 2014). The expansion of mosquito vectors (i.e., *Aedes albopictus*) to temperate regions increases the likelihood of future outbreaks (Romi et al., 2006; Fischer et al., 2013; Vega-Rua et al., 2013; Ryan et al., 2019). Since we lack both vaccines and antivirals for this arbovirus, currently vector control remains the most effective strategy to limit spread. Therefore, developing interventions that interrupt transmission is essential to mitigating the global health burden of CHIKV.

**FIGURE 1:**
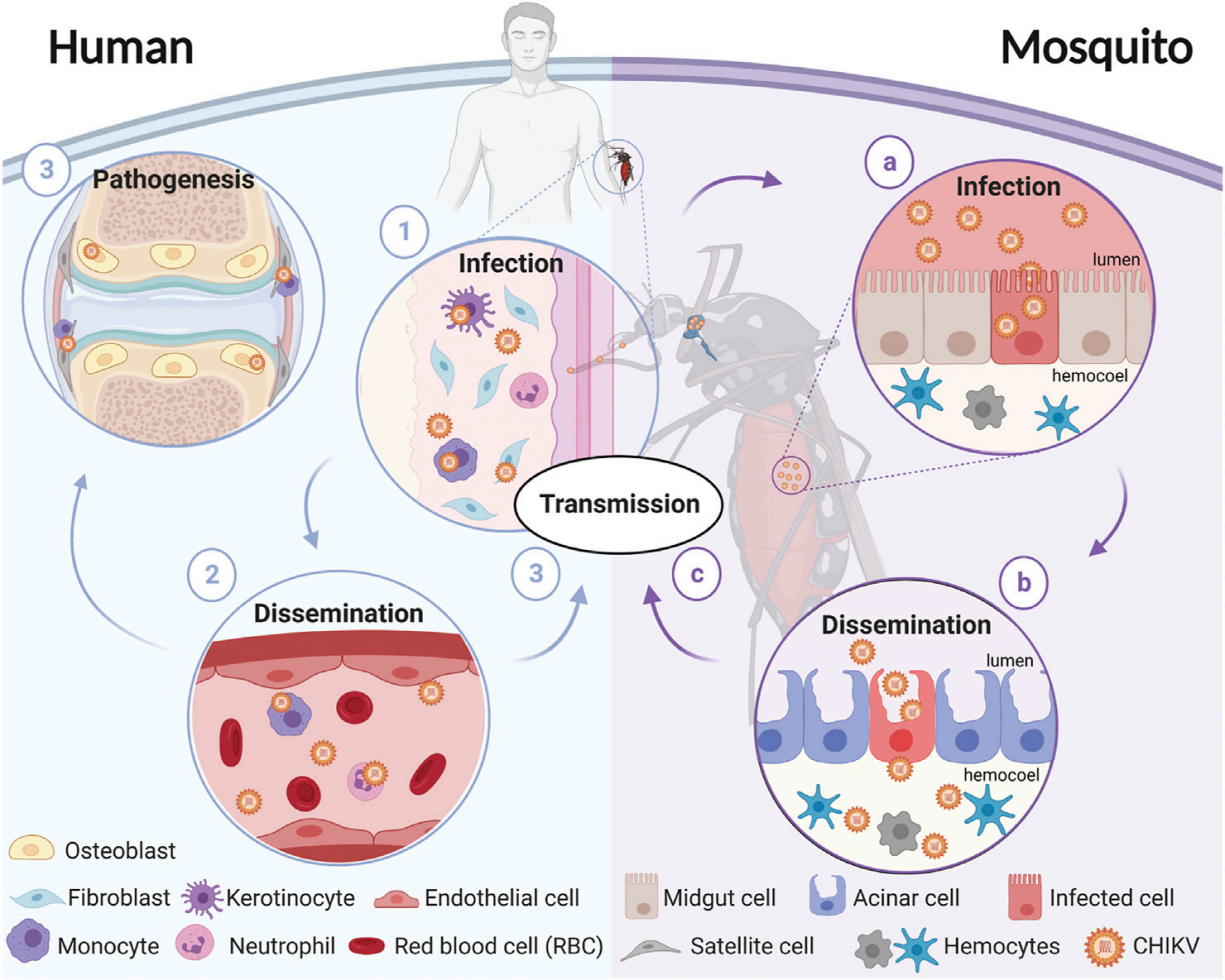
Schematic representation of CHIKV transmission cycle. CHIKV transmission starts when a female mosquito bites an infectious host. The virus enters through the bloodmeal and **(a)** infects the mosquito’s midgut, **(b)** enters the circulatory system where it disseminates to different tissues, and **(c)** eventually reaches the salivary glands. The virions present in the salivary glands of the mosquito are transmitted to a susceptible vertebrate host when the mosquito takes a blood meal. In humans, **(1)** virions enter and replicate in the fibroblasts and **(2)** disseminate until **(3)** reaching target tissues including the liver, joints, lymph nodes, muscles, and the brain. Diagram was created in biorender.com.

CHIKV, in the *Togaviridae* family, has a positive-sense single-stranded RNA genome (Simizu et al., 1984; Jose et al., 2009). Its virions are enveloped, icosahedral particles, studded with 80 glycoprotein spikes comprised of trimeric E1/E2 heterodimers (Voss et al., 2010; Sun et al., 2013). The trimeric E1/E2 spikes mediate cellular attachment (Jose et al., 2009) and fusion of viral-cellular membranes initiating infection (Kielian, 1995; Jose et al., 2009). Both cellular binding and fusion efficiently occur in mammalian and mosquito cells, suggesting that the virus must rely on highly conserved pathways or can exploit multiple pathways to enter both vertebrate and invertebrate cells. CHIKV particles interact with and productively infect a wide variety of cells, from mosquito midgut cells to human macrophages (Figure 1) (Salvador et al., 2009; Matusali et al., 2019). Matrix remodeling associated 8 (Mxra8) (Zhang et al., 2018), glycosaminoglycans (GAGs) (Tanaka et al., 2017; Weber et al., 2017; Sahoo and Chowdary, 2019; McAllister et al., 2020), C-type lectins (Prado Acosta et al., 2019; Bucardo et al., 2020) and phosphatidylserine receptors (PSRs) (Moller-Tank et al., 2013; Carnec et al., 2016; Kirui et al., 2021) have all been implicated in promoting CHIKV entry into mammalian cells. The CHIKV-Mxra8 interaction has been linked to pathogenesis (Zhang et al., 2018; Zhang et al., 2019). While Mxra8-deficient mice did not develop joint inflammation, infectious virus was still detected in peripheral tissues during acute infection (Zhang et al., 2019), supporting the notion that Mxra8 plays a role in pathogenesis, but alternative surface molecules are involved in mediating viral establishment and dissemination.

Glycosaminoglycans (GAGs) are repeating chains of negatively charged polysaccharides present on cell surfaces and in the extracellular matrix (Höök et al., 1984). GAGs, such as heparan sulfate (HS), are associated with common cellular processes including mediating adhesion and growth factor signaling (Sarrazin et al., 2011). Many viruses interact with GAGs, linking viral particles to the cell surface (Byrnes and Griffin, 1998; Shukla et al., 1999; Watterson et al., 2012). Previously, both CHIKV and Sindbis, a closely related alphavirus, were shown to utilize GAGs for attachment to host cells (Byrnes and Griffin, 1998; McAllister et al., 2020). While tissue culture adaptation can select for increased GAG interaction, some field isolates of CHIKV are associated with HS utilization (Silva et al., 2014). Production of mucopolysaccharides is conserved among vertebrates and invertebrates including human and mosquito cells (Cássaro and Dietrich, 1977; Dinglasan et al., 2007; Sinnis et al., 2007; Nakato and Li, 2016).

Phosphatidylserine receptors (PSRs) facilitate pathogen attachment to cells by binding to virion lipids (Meertens et al., 2012; Jemielity et al., 2013; Moller-Tank et al., 2013; Richard et al., 2015; Brouillette et al., 2018; Zhang et al., 2020). Viruses containing phosphatidylserine (PS) in the outer leaflet of the viral envelope can engage PSRs on host cells, mimicking apoptotic bodies and trigger internalization in a process termed apoptotic mimicry (Amara and Mercer, 2015). The production of TIMs, TAMs, or CD300a PSRs facilitates entry of Ebola, Dengue, and CHIKV in some cell lines (Shimojima et al., 2006; Meertens et al., 2012; Richard et al., 2015; Carnec et al., 2016; Dejarnac et al., 2018; Zhang et al., 2020; Kirui et al., 2021). Although the process of apoptotic mimicry in mammalian cells is well studied, the use of PSRs in mosquito cells by arboviruses is not.

Previous studies suggested that heat shock cognate 70 protein (HSC70) and ATP synthase *β* (ATPSβ) are important for CHIKV to enter mosquito cells (Fongsaran et al., 2014; Ghosh et al., 2017), but confirmatory support is lacking. Currently, no binding partner has been identified as essential for CHIKV infection. The broad host and cellular tropism of CHIKV may stem from its ability to bind a multitude of molecules present on the cell surface as opposed to a single ubiquitous factor.

In this study, we assessed the role of proposed CHIKV binding factors in both mammalian and mosquito cells. CHIKV entry into mammalian cells was highly conditional to the cell line examined. CHIKV attachment on mosquito cells does not rely on HS, PSRs, Mxra8, HSC70, or ATPSβ. Productive infection of CHIKV was not reliant on any one attachment factor. Overall, these data suggest that CHIKV entry requires an additional receptor yet to be identified or CHIKV entry can occur through a variety of cellular interactions that result in particle internalization.

## 2 Materials and methods

### 2.1 Cell lines

Human near-haploid cells (HAP1) derived from the male chronic myelogenous leukemia cell line KBM-7 (RRID:CVCL_Y019), HAP1 flippase subunit knockout (KO) line (HAP1ΔCDC50a, HZGHC005423c007, RRID:CVCL_TS94), and HAP1 scramblase KO line (HAP1ΔXKR8, HZGHC005916c007, RRID:CVCL_TY32) were purchased from Horizon Discovery (United Kingdom). HAP1 and HAP1 KO lines were cultured in Iscove’s modified Dulbecco’s medium (IMDM) supplemented with 8% (v/v) fetal bovine serum (FBS). Vero-SLAM cells (VeroS) produce the human measles receptor SLAM (Ono et al., 2001). The Vero wildtype, Vero lacking T-cell immunoglobulin mucin domain-1 (VeroΔTIM), Vero lacking Axl receptor tyrosine kinase (VeroΔAxl), and Vero lacking both TIM-1 and Axl (VeroΔTIM/Axl) cells were a kind gift from Dr. Wendy Maury at the University of Iowa (Brouillette et al., 2018). All vervet monkey cells (VeroS, VeroSΔCDC50a, VeroSΔXKR8, Vero, VeroΔTIM, VeroΔAxl, and VeroΔTIM/Axl) were maintained with DMEM supplemented with 5% (v/v) FBS. Mouse embryo fibroblast cells (NIH3T3) were purchased from ATCC (CRL-1658, RRID:CVCL_0594) and maintained with DMEM supplemented with 10% (v/v) FBS. All mammalian cells were kept in a humidified chamber held at 37°C and with a 5% CO_2_ content. Mosquito *Aedes albopictus* C6/36 (ATCC Cat# CRL-1660, RRID:CVCL_Z230) were maintained with Leibovitz’s L-15 medium supplemented with 10% (v/v) FBS in a humidified chamber held at 28°C. *Aedes aegypti* Aag2 larval homogenate cells, a kind gift from Michael Strand at the University of Georgia, were maintained in SFX insect medium with 2% (v/v) FBS in a humidified chamber at 28°C. All cell lines were periodically tested for *mycoplasma* using the PlasmoTest™—*Mycoplasma* Detection Kit (InvivoGen, cat. rep-pt1).

### 2.2 CRISPR-Cas9 mediated generation of VeroS KO cell lines

VeroS cells have a significantly higher transfection efficiency than Vero cells and were therefore chosen to produce the knockout cell lines. Three guide RNAs targeting each *Chlorocebus sabaeus* gene, XKR8 (GGC​ACT​GCT​CGA​CTA​CCA​CC, TGA​TCT​ACT​TCC​TGT​GGA​AC, CAG​CTA​TGT​GGC​CCT​GCA​CT) and CDC50a (TAC​GGC​TGG​CAC​GGT​GCT​AC, TCG​TCG​TTA​CGT​GAA​ATC​TC, GTG​AAC​TGG​CTT​AAA​CCA​GT), were inserted into pSpCas9(BB)-2A-GFP (pX458), which was a gift from Feng Zhang (Addgene plasmid #48138, RRID:Addgene_48138) (Ran et al., 2013) and verified using Sanger sequencing. VeroS cells were transfected with equivalent amounts of pSpCas9(BB)-2A-GFP bearing each of the three guide RNAs using GeneJuice (Sigma-Aldrich, cat. 70967). Three days post-transfection, VeroS cells were counted and distributed at a density of 0.5 cells per well into 96-well plates. Cells were monitored for 3 weeks to maintain single colony clones, and non-clonal wells were discarded. Wells corresponding to single clones were expanded to 24-well plates and assessed for CRISPR knockout. CRISPR XKR8 and CDC50a KOs were validated by extracting total DNA and PCR amplifying the guide RNA targeted regions. PCR amplicons spanning *xkr8* CRISPR regions were gel purified and submitted for Sanger sequencing to verify *xkr8* modification, which showed a 136 bp deletion in exon 2. We could not amplify *cdc50a* CRISPR regions in exons 1 and 3 but could amplify the CRISPR region targeting exon 5, indicating the presence of a large deletion spanning multiple exons in CDC50a. CRISPR CDC50a KO was also validated using a functional screen for externalized PS.

### 2.3 DNA transfections

Transfection efficiency and cytotoxicity varied with each cell line and gene KO. We paired different transfection reagents with different cell lines to optimize transfection efficiency and reduce cytotoxicity. HAP1 cells were transfected with JetOptimus (PolyPlus, cat. 117-07), while VeroS and 293T cells with GeneJuice (Sigma-Aldrich, cat. 70967) according to manufacturer recommendations. Expression vectors encoding a GFP-fused transmembrane hTIM-1 (a gift from Wendy Maury at the University of Iowa) (Brouillette et al., 2018), pCS6-L-SIGN (Transomic; cat. BC038851), pTiger-Mxra8 (Mxra8 open reading frame from Transomic; cat BC006213 was cloned into the pTiger expression vector), or pMax-GFP (Lonza) were used to assess CHIKV entry. pTiger was a gift from Garry Nolan (Addgene plasmid # 1728; http://n2t.net/addgene:1728; RRID: Addgene_1728).

### 2.4 Viruses

Chikungunya virus (CHIKV) strain 181 clone 25 (181/c25) was used to conduct experiments in a BSL2 laboratory environment. Reporter genes were cloned into pSinRep5-181/c25, a gift from Terence Dermody (Addgene plasmid #60078), using overlapping PCR. The reporters were added as additional transcriptional units between the non-structural and structural genes, similar to the previously characterized viruses (Tsetsarkin et al., 2006). Full-length DNA CHIKV clones containing reporter genes (*gfp, mKate, or Nluc*) were linearized and *in vitro* transcribed (Ambion, cat. AM1344) adhering to the manufacturer’s protocol. Infectious CHIKV virions encoding reporter genes were recovered after direct RNA transfection (1 µg) into VeroS cells with Lipofectamine 3000 (ThermoFisher, cat. L3000001). Unless otherwise stated, viral stocks were propagated in VeroS cells, and passage three viral stocks were used for all experiments. The amount of infectious virus was determined by calculating the 50% tissue culture infective dose (TCID_50_) units per mL through end-point dilution using the Spearman-Karber method (Ramakrishnan, 2016). Recombinant vesicular stomatitis viruses containing the CHIKV, Ebola, or Lassa glycoproteins were generated as previously described (Lay Mendoza et al., 2020). Coxsackie B5 virus was amplified in VeroS cells.

### 2.5 Cell surface staining

293T, VeroS, HAP1 and Vero cells were plated at 2 × 10^5^ cells per well in 12-well plates 48 h before staining. Cells were transfected with plasmids encoding Mxra8, hTIM-1-GFP, or L-SIGN along with a plasmid encoding GFP 24 h before immunofluorescence staining. Transfected cells were rapidly cooled and stained in blocking solution (dPBS with 1% (v/v) bovine serum albumin (BSA)) containing anti-Mxra8 (1:100, W040-3, MBL International, RRID:AB_2801291), anti-hTIM1 (1:100, AF1750, R&D Systems, RRID:AB_2116561), or anti-CLEC4M (L-SIGN/CD299) 2G1 antibody (1:100, MA5-21012, ThermoFisher, RRID:AB_2605445) at 4°C with gentle shaking for 1 h. Cells were washed with PBS before lifting the cells with a scraper. Cells were pelleted (500xg for 5 min), resuspended, and washed in PBS two additional times before adding secondary anti-goat Cy5 (1:2500, 072-02-13-06, KPL) or anti-mouse Alexa Fluor 647 (1:2500, A32728, Invitrogen, RRID: AB_2633277) and incubated at 4°C in the dark for 30 min. Cells were washed with PBS three times and then analyzed *via* flow cytometry. Cell populations were gated using forward scatter/side scatter. The mean fluorescence intensity (MFI) of the indicated secondary antibody (Cy5 or AF647) was recorded from a minimum of 10,000 GFP-positive cells per experiment. All experiments were completed three independent times. All cells were analyzed using a NovoCyte Quanteon (Agilent) flow cytometer.

### 2.6 Luminescence entry assay

Cells were plated at a density of 2.5 × 10^4^ cells per well in a 96-well plate, the day before infection. Cells were infected with CHIKV-Nluc, rVSVΔG-LASV-NlucP, or Coxsackie B5 at an MOI of 0.05. Two hours post-infection, cells were treated with 10 mM ammonium chloride (NH_4_Cl) to inhibit subsequent rounds of replication. Eight hours after infection, cells infected with CHIKV or rVSVΔG-LASV were lysed with NanoGlo substrate, and lysates were quantified in a GloMax Explorer (Promega) according to the manufacturer’s instructions. Cells infected with Coxsackie B5 were harvested 24 h post-infection and cell viability was assessed using Cell Titer Glo and quantified in a GloMax Explorer (Promega) according to the manufacturer’s instructions.

### 2.7 Competition assays

CHIKV-Nluc stocks were used to assess the ability of antibodies, liposomes, heparan sulfate, or sodium azide to block infection in the indicated cell lines. Cells were seeded at 5 × 10^4^ cells per well in a 96-well plate, 1 day prior to infection. For each well in the competition assay, approximately 150 CHIKV-Nluc virions were added. 24 h following infection, cells were lysed with NanoGlo substrate, and lysates were quantified in a GloMax Explorer (Promega) according to the manufacturer’s instructions. Data is displayed as percent of control which was calculated by dividing the luminescence values at each condition with the control (mock inhibitor added).

*Antibody competition to the virus*: Virus was incubated with the indicated concentrations of CHIKV polyclonal antibody (IBT, cat. 04-008) or no antibody control (PBS) at room temperature for 45 min. After incubation, the virus-CHIKV antibody mix was added to the cells.

*Antibody and sodium azide competition to the cells*: Cells were incubated with the indicated concentrations of Mxra8 clone 9G2. D6 antibody (EMD Millipore Corp., cat. MABF2275), HSC70 monoclonal antibody (Invitrogen, cat. MA3-014, RRID:AB_325462), Mouse IgG2a K Isotype control eBM2a (eBioscience Inc., cat. 14-472481), ATP5B pAb (Abnova, cat. H00000506-D01P, H00000506-D01P), XKR8 antibody (ThermoFisher, cat. PA5-65799), human EBOV monoclonal KZ52 antibody (IBT, cat. 0260-001), sodium azide (Sigma-Aldrich, cat. 26,628-22-8), or control (PBS). After incubation at 37°C for 20 min, cells were infected.

*Heparan Sulfate competition*: Following a protocol previously found to block CHIKV infection in CHO cells (Silva et al., 2014), the indicated concentration of heparan sulfate (Sigma-Aldrich, cat. H7640-1 mg), or control PBS was added to cells at 37°C for 10 min prior to infection. After the 10 min pre-treatment, virus was added.

*Liposome competition*: PC:PE:PS liposomes (75% PC: 20% PE: 5% PS) and PC-only liposomes (Zhang et al., 2020), were sonicated for 20 min or 1 h respectively. The indicated concentration of liposomes or PBS was added to cells at 37°C for 10 min prior to infection. After the 10 min pre-treatment, virus was added.

### 2.8 Cell-to-cell viral spread kinetics

Cells were plated at either 7.5 × 10^4^ cells per well in a 48-well plate (HAP1 lines) or 5 × 10^4^ cells per well in a 24-well plate (Vero lines) 1 day prior to infection. Assuming the density of cells doubled overnight, cells were inoculated with CHIKV-GFP virus (MOI of 0.1). After 1 h (T = 0 hpi) virus inoculum was removed and replaced with complete media. At the indicated time, cells were lifted in trypsin, resuspended in PBS, and fixed in 1.85% (v/v) formaldehyde. GFP-positive cells were enumerated in a NovoCyte Quanteon (Agilent) flow cytometer. Live cells were first gated based on forward/side scatter, and cellular aggregates were removed by gating with forward scatter area to height. Uninfected cells were used to set the GFP gate. 10,000 live cells were collected and the percent infection (% GFP^+^) was recorded and compared over time.

### 2.9 Luminescence entry kinetics assay

Vero and ΔTIM/Axl cells were seeded at 2.5 × 10^4^ cells per well in a 96-well plate. One day after seeding, cells were infected with CHIKV-Nluc with approximately 500 or 50 CHIKV-Nluc virions per well, as indicated. NH_4_Cl at 10 mM was added at the indicated time points to inhibit low pH in the endosomal compartments. 8 h post-infection, cells were lysed with NanoGlo substrate, and lysates were quantified in a GloMax Explorer (Promega) according to the manufacturer’s instructions.

### 2.10 Quantification of cellular outer leaflet phosphatidylserine (PS)

Cellular surface levels of PS were assessed using Promega’s RealTime-Glo Annexin V Apoptosis and Necrosis Assay (Promega, cat. JA1012) according to manufacturer specifications. HAP1 or VeroS cell lines were plated in media supplemented with 0.1 M HEPES at 3.0 × 10^4^ or 10^4^ cells per well, respectively, in a 96-well black-walled, clear bottom plate one day before treatment. Cells were infected with CHIKV-mKate (MOI of 1.0) or mock infected. Kit components 1–4 were added to cells 1 h following infection and the plate was moved into a pre-warmed GloMax Explorer. Kit components 1–4 were used at 0.5x concentration in HAP1 cell lines as cytotoxicity was observed at 1x manufacturer recommendations. Luminescence (Annexin V) measurements were collected 24 h following infection in a GloMax Explorer (Promega) held at 37°C.

### 2.11 Real-time quantification PCR (RT-qPCR) of genome equivalents

CHIKV genome equivalents/mL were calculated *via* RT-qPCR. Viral RNA was extracted from infected cell supernatant (Zymo, cat. 11–355), eluted in nuclease-free water, and converted to cDNA with random hexamer primers (ThermoFisher, cat. 4388950) following kit protocols. RT-qPCR reactions were set up with cDNA, TaqMan Gene Master Mix (Applied Biosystems, cat. 4369016), primers, and TaqMan probe (5′-6FAMACTTGCTTTGATCGCCTTGGTGAGAMGBNFQ-3′) as previously described (McCarthy et al., 2018) with each sample run in duplicate. A plasmid-based standard curve of a full-length CHIKV clone was used to enumerate the total number of genome equivalents per mL of the original sample. A no template control (NTC) and no amplification control (NAC) were included in each run on a StepOne platform (Applied Biosystems, cat. 4376357). The amplification profile included 1 cycle of 2 min at 50°C, 10 min at 95°C, followed by 40 cycles of 15 s at 95°C and 1 min at 60°C.

### 2.12 Quantification of viral outer leaflet phosphatidylserine (PS)

*Virus Production:* T75 flasks were seeded with wild type, ΔXKR8, and ΔCDC50a HAP1 and VeroS cells with 7.2 × 10^6^ cells or 3.6 × 10^6^ cells, respectively. After 24  h, wild-type and ΔXKR8 cells were infected with CHIKV using an MOI of 0.001, and ΔCDC50a cells were infected using an MOI of 0.01. After 12 h at 37°C, the inoculum was removed, cells were treated with citric acid buffer [40 mM citric acid, 10 mM KCl, 135 mM NaCl (pH 3.0)] for 1 min, rinsed, and FBS-free media was added. After incubating for an additional 36 h, the supernatant was collected, cleared twice using centrifugation (6,000 × g), and overlaid on a 20% sucrose cushion. Overlaid supernatants were then subjected to ultracentrifugation at (234,116 × g) for 2 h at 4°C. Pellets were resuspended in 100 μL PBS.

*Input normalization:* Prior to staining, purified CHIKV samples were normalized using RT-qPCR. To ensure normalization we compared protein levels, normalized samples were denatured using SDS-urea buffer [200 mM Tris (pH 6.8), 8 M urea, 5% SDS, 0.1 mM EDTA, 0.03% bromophenol blue], run on Mini-PROTEAN TGX Stain-Free Precast Gels (Bio-Rad), and imaged with a ChemiDoc XRS digital imaging system (Bio-Rad), capsid protein was readily detected. Gels were then subjected to immunoblot analysis for CHIKV E using an anti-E antibody (1:1000, R&D Systems, MAB97792SP).

*Particle surface PS staining:* Similar to previous protocols (Nanbo et al., 2018; Acciani et al., 2021), equivalent numbers of CHIKV particles were conjugated to 4-μm aldehyde/sulfate latex beads (ThermoFisher) overnight at 4°C with gentle shaking. Due to differences in viral yields between cell lines, beads were bound with approximately 10^6^ genome equivalents from HAP1 cell lines and 10^9^ genome equivalents from VeroS cell lines.

Beads were blocked with a final concentration of 1% (v/v) bovine serum albumin (BSA) in PBS for 2 h while rotating at room temperature. Beads were washed 3 times with 1% (v/v) BSA in PBS and then incubated with 100 μL of AnV binding buffer containing AnV-PE conjugate for 30 min on ice. Beads were diluted 1:4 in AnV binding buffer and analyzed using the NovoCyte Quanteon flow cytometer (Agilent). Bead-only samples were included as a mock control.

### 2.13 Specific infectivity

We used the ratio of infectious viral particles to genome equivalents to assess particle infectivity. This ratio represents the number of infectious particles in a viral stock. A value close to 1 indicates a virus stock is more infectious, or each particle has a higher probability of starting an infection. Particle number was determined by quantifying the number of genome equivalents in the virus preparation using qRT-PCR described above. When comparing various cell lines, infectivity was determined by TCID_50_ units per mL, instead of the traditional plaque forming units (PFUs) as not all our cell lines tolerated forming a confluent monolayer under an agar.

### 2.14 PNGase F and heparinase digestion

Ultracentrifuge-concentrated CHIKV-Nluc virions were treated with PNGase F (New England Biolabs, cat. P0704S) or Heparinase II from *Flavobacterium heparinum* (Sigma-Aldrich, cat. H6512) in a 37°C water bath for 18 h following manufacturer’s non-denaturing protocol. An aliquot of treated virions was denatured and analyzed through SDS-PAGE using an anti-CHIKV E1 antibody (1:1000, R&D Systems, MAB97792SP). To assess the effect of treatment on infection, cells were plated at 2.5 × 10^4^ cells per well in a 96-well plate. Cells were infected at an MOI of 0.05 for 24 h, lysed with NanoGlo substrate, and quantified in a GloMax Explorer (Promega) according to the manufacturer’s instructions.

### 2.15 Sodium azide cell viability assays

Cells were seeded at 5 × 10^4^ cells per well in a 96-well plate, 1 day prior to infection. Sodium azide (Sigma-Aldrich, cat. 26628-22-8), or control (PBS) was added to each well and incubated at 37°C for 20 min. Following incubation, approximately 150 CHIKV-Nluc virions were added to each well. 24 h following infection, cells were lysed with Cell Titer Glo or RealTime-Glo MT (Promega), and lysates were quantified in a GloMax Explorer (Promega) according to the manufacturer’s instructions. Data is displayed as percent of control which was calculated by dividing the luminescence values at each condition with the control (mock inhibitor added).

### 2.16 Flow cytometry entry assay

Cells were plated at 2.5 × 10^5^ cells per well in a 24-well plate, 1 day prior to infection. CHIKV-GFP virus inoculum was prepared using DMEM FBS-free media and 250 μL was added to each cell line. After 2 h, the virus inoculum was removed and replaced with complete media containing NH_4_Cl (10 mM). 18hpi, cells were lifted, resuspended in PBS, and fixed in 1.85% (v/v) formaldehyde. GFP-positive cells were enumerated in a NovoCyte Quanteon (Agilent) flow cytometer. Live cells were first gated based on forward/side scatter, and cellular aggregates were removed by gating with forward scatter area to height. Uninfected cells were used to set the GFP gate. At least 10,000 live cells were collected and the percent infection (% GFP^+^) was recorded.

### 2.17 Statistical analysis

Data were visualized and analyzed using GraphPad Prism software (v9.4.0, macOS). An unpaired parametric Student’s t-test assuming equal variance was used to test for statistical significance for data on a linear scale (e.g., percent GFP^+^). An unpaired parametric Student’s t-test using a Welch’s correction was used to test for statistical significance for normalized data (e.g., percent of control, normalized MFI). Logarithmic data were natural log (ln) transformed and then assessed with an unpaired parametric Student’s t-test assuming equal variance (e.g., titer, luminescence). When comparing all cell lines, ANOVA with Tukey’s multiple comparisons test was used.

## 3 Results

### 3.1 Exogenously expressed attachment factors differentially enhance CHIKV infection

Previous studies demonstrate that CHIKV infection can be enhanced by the addition of proteins including Mxra8, C-type lectins (i.e., L-SIGN), and PSRs (i.e. TIM-1 and Axl) in 293T cells (Jemielity et al., 2013; Moller-Tank et al., 2013; Kirui et al., 2021). Human 293T cells are an epithelial-like cell isolated from a fetal kidney (Graham et al., 1977). While 293T are permissive for CHIKV infection, they are poorly susceptible, and the addition of various attachment factors enhances entry (Jemielity et al., 2013; Moller-Tank et al., 2013; Kirui et al., 2021). First, we confirmed previous findings in 293T cells and expanded the evaluation to include HAP1 and VeroS cells (Carnec et al., 2016; Roberts et al., 2017; McAllister et al., 2020). HAP1 cells, a human haploid cell line derived from chronic myeloid leukemia KBM7 cells, have been used to understand CHIKV interactions with GAGs (Tanaka et al., 2017; McAllister et al., 2020). Vero cells are a vervet monkey kidney cell line that is commonly used to amplify viral stocks (Kiesslich and Kamen, 2020). VeroS cells are Vero cells engineered to produce the measles virus receptor SLAM. VeroS cells are readily transfected whereas Vero cells are not, therefore in experiments requiring transfection, VeroS cells were used. Antibody staining and flow cytometry was used to establish the presence or absence of endogenous surface TIM-1, Mxra8, or L-SIGN in the cell lines (Supplementary Figure S1). Aligned with published data, 293T and HAP1 cells lack the endogenous surface presentation of TIM-1 (Kondratowicz et al., 2011; Meertens et al., 2012; Jemielity et al., 2013; Kirui et al., 2021), Mxra8 (Zhang et al., 2018; McAllister et al., 2020), and L-SIGN (Lozach et al., 2003), while endogenous TIM-1 and Mxra8 are present on the surface of VeroS cells (Supplementary Figure S1).

**FIGURE 2:**
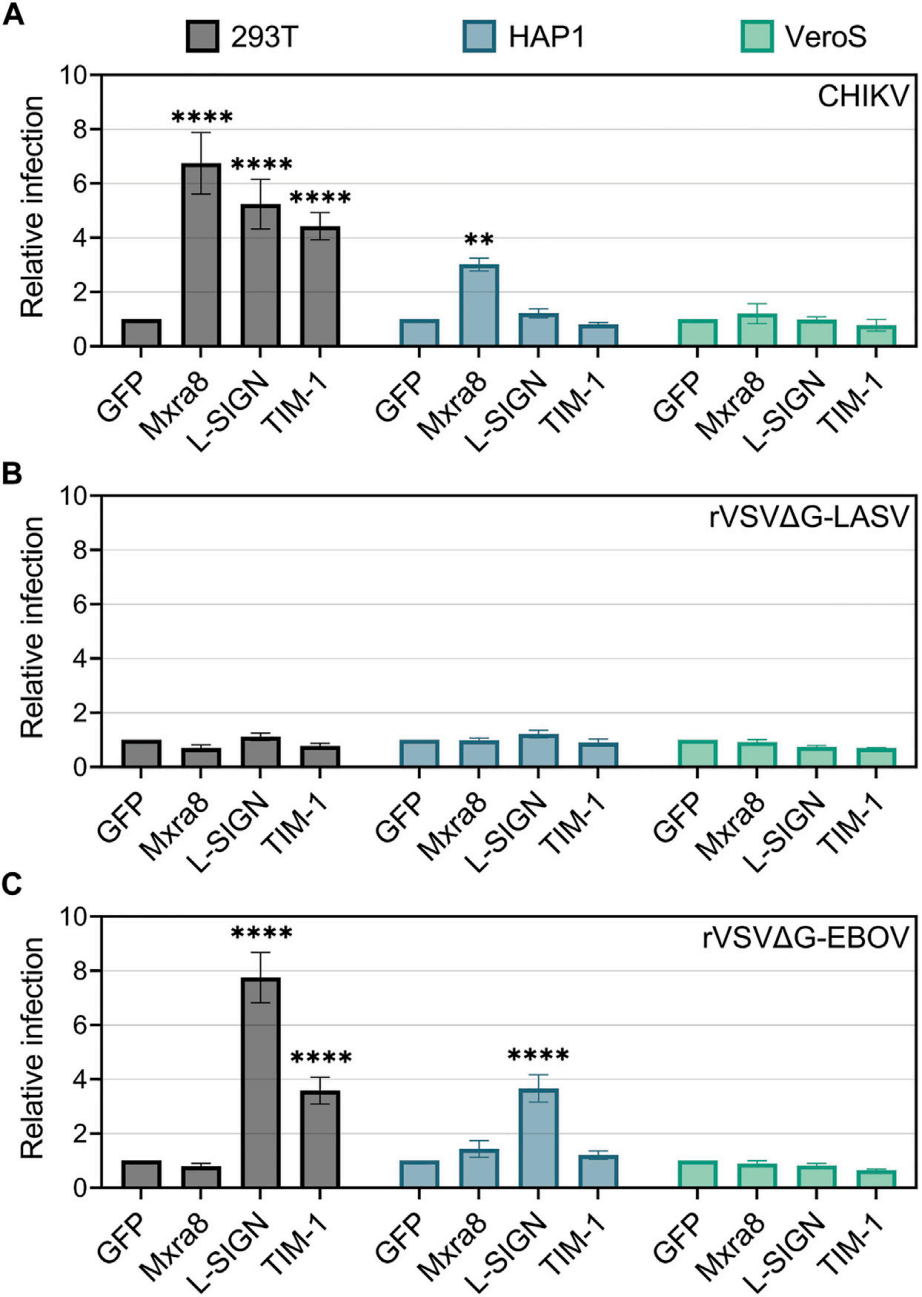
Mxra8, L-SIGN, and TIM-1 enhance CHIKV infection in 293T cells. 293T, HAP1, and VeroS cells were transfected with either TIM-1-GFP, Mxra8 and GFP, L-SIGN and GFP, or GFP alone. 24 h post-transfection, cells were inoculated with either mKate-expressing **(A)** CHIKV strain 181/c25, **(B)** recombinant vesicular stomatitis virus containing the Lassa virus glycoprotein (rVSVΔG-LASV), or **(C)** rVSV containing the Ebola virus glycoprotein (rVSVΔG-EBOV) for 1 h. 12 h following infection the cells were enumerated in a flow cytometer. Relative infection was calculated as the proportion of infected cells (mKate^+^) among transfected cells (GFP^+^) normalized to infection levels in a GFP-only control. Data are presented as the mean ± SEM from three independent experiments. Data were compared with a two-way ANOVA with multiple comparisons, comparing row effects (receptors produced) to the GFP only control: **, *p* < .01; ****, *p* < .0001.

To determine if the over-expression of TIM-1, Mxra8, and/or L-SIGN can enhance CHIKV infection, cells were transfected with plasmids encoding each entry factor either fused to GFP or along with a plasmid encoding GFP. Plasmid transfection effectively produced the entry factors on the cell surface (Supplementary Figure S1). In 293T cells, exogenous Mxra8, TIM-1, and L-SIGN all similarly enhanced CHIKV infection, while only Mxra8 promoted infection in HAP1 cells (Figure 2A). Over-expression of Mxra8 and TIM-1 or introducing L-SIGN in VeroS cells did not enhance CHIKV entry.

To confirm that the infection enhancements were specific to CHIKV, we infected cells producing exogenous entry factors with recombinant vesicular stomatitis virus (rVSV) containing the Lassa virus (rVSVΔG-LASV) (Figure 2B) or Ebola virus (rVSVΔG-EBOV) (Figure 2C) glycoproteins (Lay Mendoza et al., 2020). Both 293T and HAP1 cells produce properly glycosylated alpha-dystroglycan, the high-affinity receptor for Lassa virus (Cao et al., 1998; Jae et al., 2013), whereas VeroS cells do not (Kunz et al., 2005; Brouillette et al., 2018). As expected, the overproduction of TIM-1, Mxra8, or L-SIGN did not significantly increase rVSVΔG-LASV infection in either 293T, HAP1, or VeroS cells (Figure 2B).

Entry of rVSVΔG-EBOV is enhanced by PSRs (Jemielity et al., 2013; Moller-Tank et al., 2013) and L-SIGN (Alvarez et al., 2002; Takada et al., 2004), but not by Mxra8. As expected, rVSVΔG-EBOV infection was enhanced in TIM-1^+^ 293T cells but Mxra8 did not enhance infection in any of the cell lines (Figure 2C). L-SIGN enhanced rVSVΔG/EBOV infection by 8-fold in 293T cells and 3.5-fold in HAP1 cells but had no effect in VeroS cells (Figure 2C). Together, these data suggest the various attachment factors can all enhance CHIKV entry, but in specific cell lines.

### 3.2 CHIKV exhibits a cell line-dependent use of entry factors in mammalian cells

**FIGURE 3:**
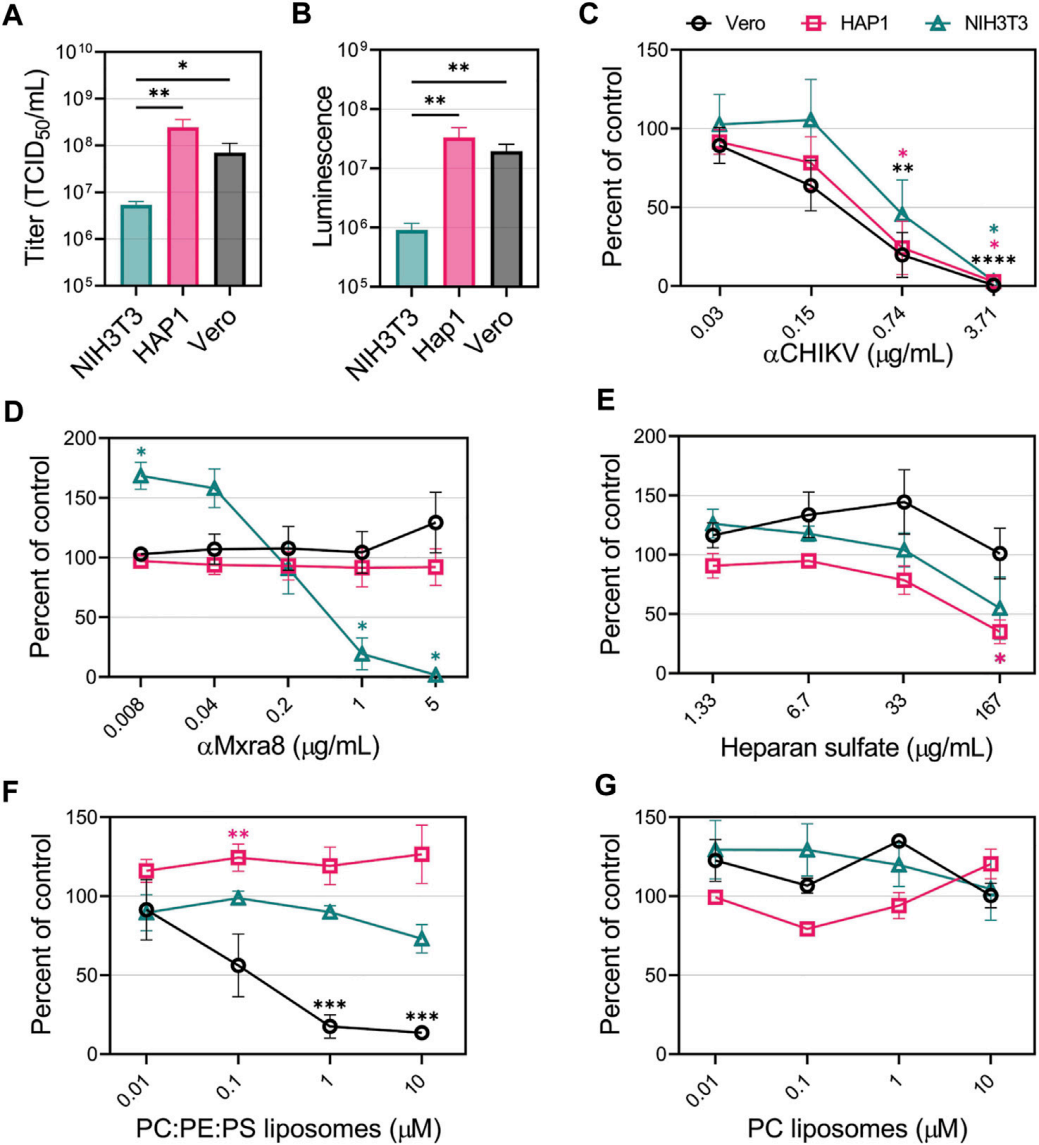
CHIKV viral apoptotic mimicry is cell-type dependent in mammalian cells. **(A)** CHIKV stock titers NIH3T3, HAP1, and Vero cells. **(B)** Luminescence levels produced by CHIKV-Nluc in NIH3T3, HAP1, and Vero cells after one round of replication. CHIKV-Nluc stocks were used to assess the ability of **(C)** CHIKV antibody, **(D)** Mxra8 antibody, **(E)** heparan sulfate, **(F)** PS-containing liposomes (75% PC: 20% PE: 5% PS), and **(G)** PC-only liposomes, to block infections into either NIH3T3 (△), HAP1 (□), or Vero (◯) mammalian cells at the indicated concentrations. Data are presented as the mean percent of control ± SEM from at least three independent experiments performed in duplicate or triplicate. For each treatment, the level of Nluc was measured and compared to the no inhibitor added control to determine percent of control. **(A,B)** Unpaired parametric Student’s t-test was performed to determine statistical significance among cell lines **(C–E)** with unequal variance (Welch’s correction) compared to a no-treatment control, or **(F,G)** comparing between PC:PE:PS vs. PC liposomes. *, *p* < .05; **, *p* < .01; ***, *p* < .001; ****, *p* < .0001.

To compare the CHIKV entry pathways in mammalian cells without over-expressing entry factors, we focused on 3 cell lines: 1) NIH3T3 cells, a mouse fibroblast cell line that was used to identify Mxra8 as a CHIKV receptor (Zhang et al., 2018); 2) HAP1 cells, and 3) Vero cells. First, CHIKV viral stocks were titrated on the 3 cell lines. We observed that HAP1 and Vero cells were the most susceptible and permissive, displaying titers 10-fold higher than the NIH3T3 cells (Figure 3A). We also monitored reporter activity produced after a single round of infection with CHIKV-Nluc. Luciferase activity mirrored the titer data, with both HAP1 and Vero cells producing more than 10-fold higher signals than NIH3T3 cells (Figure 3B). These differences suggest that the entry and/or replication efficiency of CHIKV varies among different cell lines.

To determine the relevance of GAGs, PSRs, and Mxra8 in facilitating CHIKV infection in HAP1, NIH3T3 and Vero cells, we performed competitive inhibition assays. First, to demonstrate CHIKV entry could be blocked in all cell lines, we used a neutralizing CHIKV antibody to inhibit infection. As expected, the luciferase signal produced by CHIKV-Nluc infection was reduced in a dose-dependent manner with the addition of increasing concentrations of CHIKV antibody (Figure 3C). However, increasing amounts of Mxra8 antibody only inhibited CHIKV infection in NIH3T3 cells but did not affect entry into HAP1 nor Vero cells (Figure 3D). The role of GAGs in CHIKV infection was evaluated by adding heparan sulfate (HS) to compete for entry. Soluble HS can compete with HS on the cells, blocking CHIKV entry (Silva et al., 2014; McAllister et al., 2020). High concentrations of soluble HS significantly inhibited CHIKV infection in HAP1 cells but did not negatively impact entry into Vero cells at any concentration (Figure 3E). HS modestly inhibited infection into NIH3T3 cells at the highest concentration, but the reduction was not statistically significant (Figure 3E).

To prevent interaction with PSRs, we added liposomes containing the anionic phospholipids PS and phosphatidylethanolamine (PE) dispersed in neutral phosphatidylcholine (PC), previously demonstrated to efficiently bind PSRs (Zhang et al., 2020). PC:PE:PS liposomes did not inhibit CHIKV infection in HAP1 and NIH3T3 cells (Figure 3F). Conversely, Vero cells exhibited dose-dependent inhibition, where a ∼90% reduction in infection was achieved by competing with 10 μM PC:PE:PS liposomes (Figure 3F). PC-only liposomes did not inhibit CHIKV infection in any of the cell lines (Figure 3G), confirming the PS/PE-dependent inhibition in Vero cells. These data support the hypothesis that CHIKV entry can occur through several different entry pathways, but specific pathways may mediate the majority of CHIKV entry into a given cell line: Mxra8 in NIH3T3 cells, HS in HAP1 cells, and PSRs in Vero cells.

### 3.3 CHIKV entry into Vero cells is driven mainly through PS receptors

**FIGURE 4:**
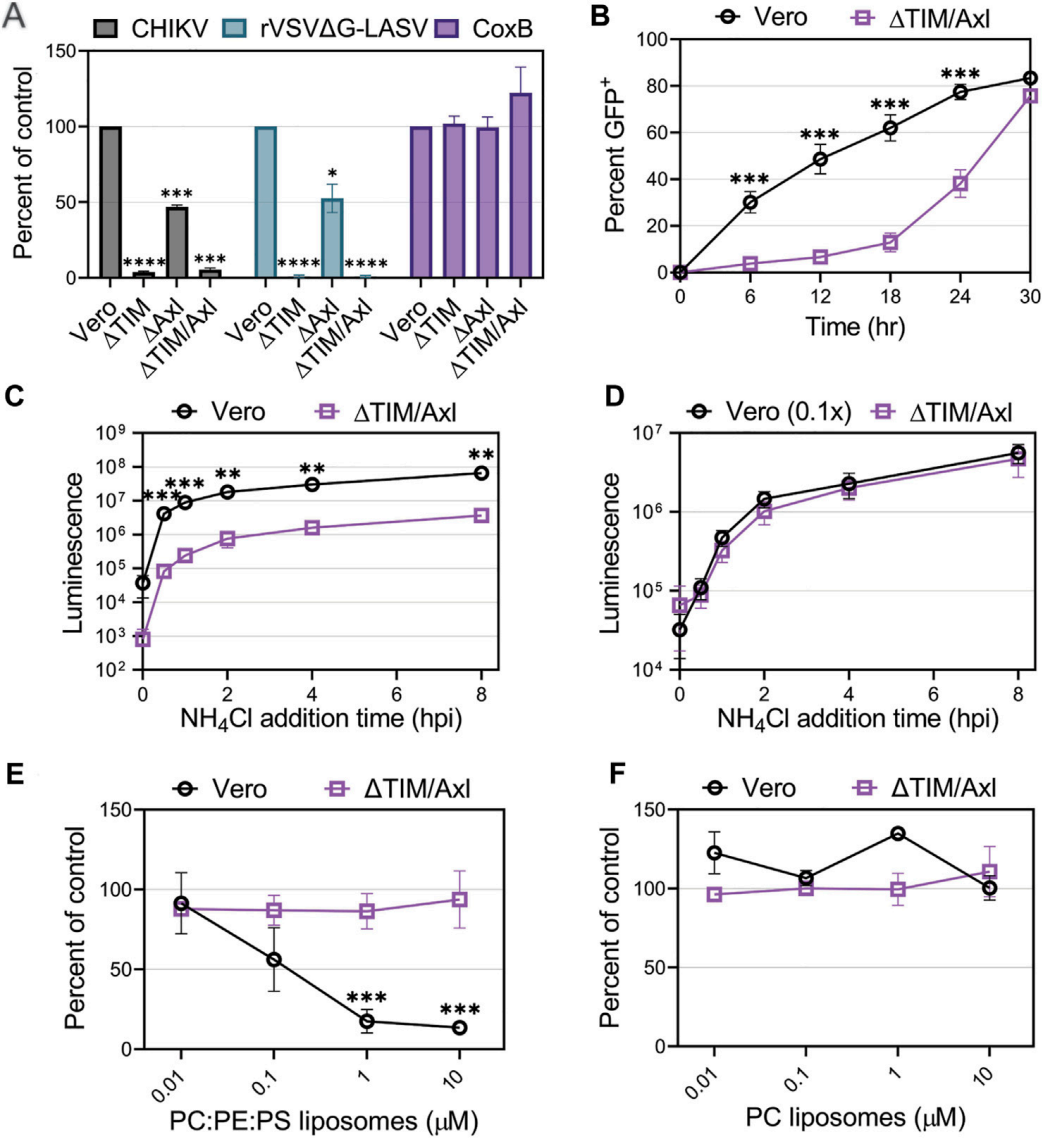
Entry of CHIKV into Vero cells is mediated mainly through TIM-1. **(A)** Entry of CHIKV-Nluc, rVSVΔG-LASV-Nluc, and CoxB into Vero, VeroΔTIM, VeroΔAxl, and VeroΔTIM/Axl after one round of replication was assessed by luciferase assays or cell viability (CoxB). **(B)** Luminescence values from the knockout cells was compared to the values observed in the parental Vero to determine the percent of control. CHIKV-GFP cellular spread kinetics were quantified by flow cytometry and the percent of GFP^+^ cells over time in Vero and VeroΔTIM/Axl cells. Kinetics of endosomal escape of CHIKV was determined by infecting Vero and VeroΔTIM/Axl cells with **(C)** equal amounts of virus or **(D)** 10-fold less virus in Vero cells. Cells were treated with NH_4_Cl at the indicated time points and luminescence was measured 8 h post-infection. The ability of **(E)** PS-containing liposomes (75% PC: 20% PE: 5% PS) and **(F)** PC-only liposomes, to block CHIKV infection into VeroΔTIM/Axl, cells was assessed at the indicated concentrations. The data for each respective panel were generated at the same time and in the same way as the data displayed in Figure 3. We present the Vero data from Figure 3 again to allow for easy visual comparison to the VeroΔTIM/Axl data. Unpaired parametric Student’s t-test was performed to determine statistical significance with unequal variance comparing PC:PE:PS to PC liposomes. Data are presented as the mean percent of control ± SEM from three independent experiments performed in triplicate. *, *p* < .05; **, *p* < .01; ***, *p* < .001; ****, *p* < .0001.

CHIKV entry into Vero cells was greatly reduced in the presence of anionic phospholipid liposomes suggesting PS receptors may play a role in mediating CHIKV entry into Vero cells. Vero cells produce both TIM-1 and Axl which bind to and internalize PS-containing cargo (Brouillette et al., 2018). To further evaluate CHIKV entry into Vero cells, we compared CHIKV-Nluc infection in Vero cells lacking TIM-1 (∆TIM), Axl (∆Axl), or both TIM-1 and Axl (∆TIM/Axl). Similar to our VeroS cells, Vero cells present endogenous TIM-1 and Mxra8 (Supplementary Figure S2). Entry of rVSVΔG-LASV in Vero cells relies mainly on TIM-1 production, but lack of Axl expression can modestly decrease susceptibility to infection (Brouillette et al., 2018). We observed that CHIKV infection closely mirrors LASV entry (Figure 4A). Cells lacking Axl displayed reduced infection (∼50%), but entry into ∆TIM or ∆TIM/Axl cells was substantially inhibited (>95%). To demonstrate that the cells retain susceptibility to other viruses, we used Coxsackie B virus (CoxB), a naked virus that enters through the Coxsackie and adenovirus receptor (CAR) (Bergelson et al., 1997). CoxB was able to infect all four Vero lines at similar efficiencies (Figure 4A).

When multi-cycle CHIKV infection was examined, we observed that, despite a delay, CHIKV eventually spreads and infects ∆TIM/Axl cells (Figure 4B). To monitor how quickly the particles are internalized in the cells and escape from the low pH cellular compartment, we blocked endosomal escape with ammonium chloride (NH_4_Cl) at multiple time points throughout infection. While the ∆TIM/Axl cells produced lower luciferase levels, the time of endosomal escape appeared similar to Vero cells (Figure 4C). To adjust for the total level of virus entering the ∆TIM/Axl cells, we added 1/10th of the virus to the parental Vero cells. This amount of virus resulted in a similar level of infection in both Vero and ∆TIM/Axl cells (Figure 4D). CHIKV appeared to escape from the endosome at the same rate in both cell lines, suggesting the lack of TIM-1 and Axl decreases particle binding or internalization efficiency, but once endocytosis is initiated, the viral particles are trafficked to a low-pH compartment at a similar rate.

To explore how CHIKV infection proceeds in Vero cells lacking TIM-1 and Axl, we assessed the role of Mxra8 antibody, heparan sulfate, or liposomes to block CHIKV infection through competition assays (Figures 4E, F and Supplementary Figures 2E, 2F). We did not observe significant virus inhibition in the ∆TIM/Axl cells by any treatment, suggesting CHIKV is utilizing an additional minor entry pathway in Vero cells in the absence of the PSRs TIM-1 and Axl.

### 3.4 Increased levels of exposed viral envelope PS increase CHIKV infectivity in Vero cells

**FIGURE 5:**
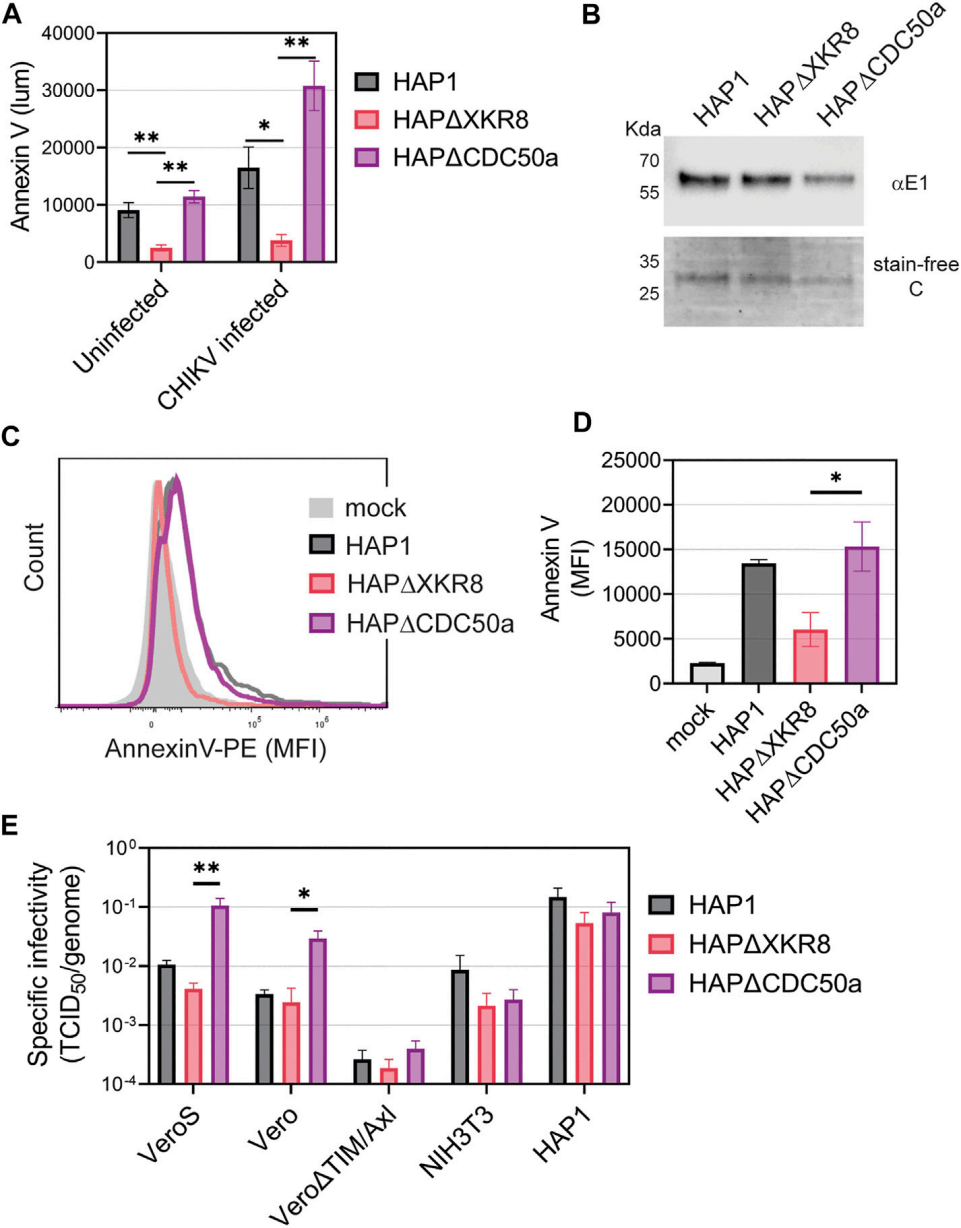
CHIKV virion PS levels correlate with specific infectivity in Vero cells. **(A)** HAP1 cell lines were monitored for Annexin V binding (luminescence) at 24 h using a GloMax Explorer microplate reader. Parental, ΔXKR8, and ΔCDC50a cells were either untreated or infected with CHIKV (strain 181/c25, MOI 1). **(B)** CHIKV was propagated in HAP1 cells and HAP1 cell lines knocked out for scramblases (ΔXKR8) and flippase subunits (ΔCDC50a). Particles were normalized to genome equivalents and examined with immunoblot using a CHIKV E1 antibody and assessed for purity using a stain-free gel. **(C,D)** Annexin V conjugated to PE was used to stain normalized amounts of virus-bound beads and quantified *via* flow cytometry. A bead-only control (mock) was used to establish a baseline signal. MFI values from three independent trials were normalized to parental values (HAP1) with the mean and ±SEM displayed. An unpaired parametric *t*-test with Welch’s correction was used test for statistical significance between ΔXKR8 and ΔCDC50a conditions. **(E)** The ratio of TCID_50_ to genome copy equivalents for each sample was used to assess the infectivity of particles produced from HAP1 cell lines on a panel of commonly used mammalian cell lines (monkey Vero, mouse NIH3T3, and human HAP1). Data are presented as the mean ± SEM from three independent experiments performed in duplicate or triplicates. Infectivity values were natural log (ln) transformed before performing an unpaired parametric Student’s t-test between ΔXKR8 and ΔCDC50a conditions. *, *p* < .05; **, *p* < .01.

To further evaluate the role of PS and PSRs in CHIKV infection, we produced CHIKV particles with either high or low levels of PS in the outer leaflet of the virion membrane. Cells maintain PS asymmetry with flippases that constitutively move PS from the outer to inner leaflet of the plasma membrane (Segawa et al., 2014; Acciani and Brindley, 2022). During cell death, which is triggered during CHIKV infection, flippases become inactive and scramblases are activated, increasing PS levels in the outer leaflet (Acciani and Brindley, 2022). By producing virus in cells with modified PS translocation dynamics, we can produce particles with either high or low levels of PS (Acciani et al., 2021). Knocking out the scramblase XKR8 in HAP1 cells (HAP1ΔXKR8) prevents apoptosis-induced scramblase activity, resulting in cells with low outer leaflet PS even during CHIKV infection (Figure 5A) (Acciani et al., 2021). In contrast, knocking out the flippase subunit CDC50a in HAP1 cells (HAP1ΔCDC50a) eliminates P4-ATPase flippase activity, resulting in cells with relatively high PS levels in the outer leaflet of the plasma membrane (Figure 5A). Particles produced in ∆XKR8 were low in PS compared to particles produced in ∆CDC50a, which were PS-high (Figures 5B–D). Since CHIKV infection induces apoptosis, we were not surprised to find that particles produced in infected HAP1 cells have PS levels at only moderately lower levels than our PS-high particles produced in HAP1ΔCDC50a cells (Figure 5D).

We infected a panel of mammalian cell lines with the PS-high and PS-low CHIKV virions to determine if the envelope PS levels altered infectivity. Genome equivalents were calculated for each viral inoculum and compared to the titer based on tissue culture infectious dose 50 value (TCID_50_) to calculate the specific infectivity on each cell line. The levels of PS on the particle correlated positively with specific infectivity when infecting Vero and VeroS cells (Figure 5E). This correlation was not observed in Vero∆TIM/Axl, NIH3T3, nor HAP1 cells (Figure 5E). Similar data was obtained when CHIKV particles were produced in VeroS cells lacking CDC50a or XKR8, except that wild-type VeroS produced particles had a PS profile more akin to particles produced in VeroSΔXKR8 cells due to weak scramblase activity (Supplementary Figure S3). This further suggests that CHIKV entry into Vero cells is driven primarily by apoptotic mimicry, while it occurs through alternative pathways in the other tested mammalian cell lines.

### 3.5 CHIKV infection of mosquito cell lines is not mediated through PS receptors or heparan sulfate

**FIGURE 6:**
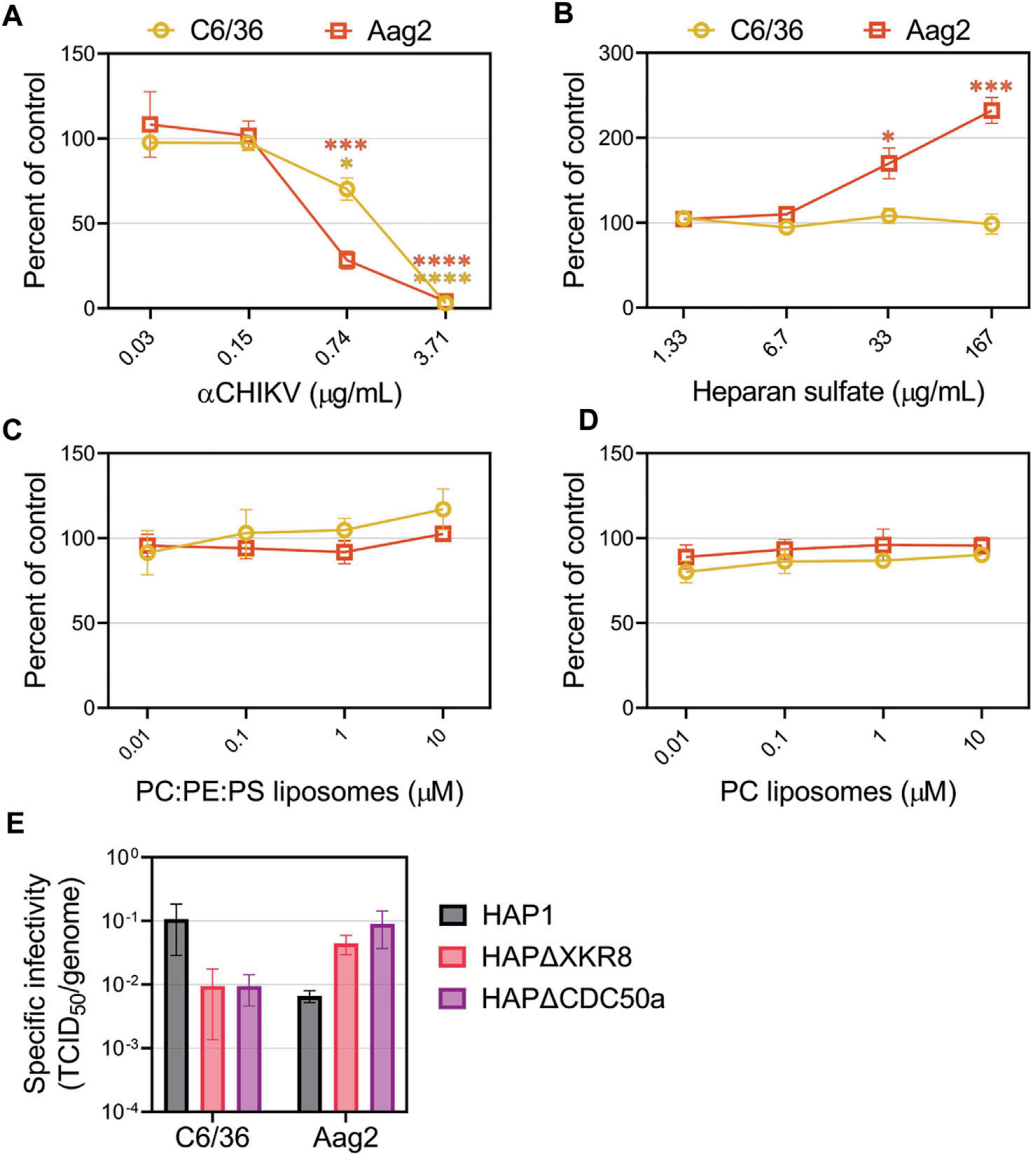
CHIKV infection in mosquito cells is not inhibited by the addition of heparan sulfate or liposomes. CHIKV stocks were used to assess the ability of **(A)** CHIKV antibody **(B)** heparan sulfate **(C)** PS-containing liposomes (75% PC: 20% PE: 5% PS) and **(D)** PC-only liposomes to block infections into either C6/36 or Aag2 mosquito cells at the indicated concentrations. **(E)** The ratio of TCID_50_ to genome copy equivalents for each sample was used to assess the infectivity of particles produced from HAP1 cell lines on mosquito C6/36 and Aag2 cells. Infectivity values were natural log (ln) transformed prior to performing an unpaired parametric Student’s t-test between ΔXKR8 and ΔCDC50a conditions. Data are presented as the mean ± SEM from at least three independent experiments performed in triplicate. *, *p* < .05; ***, *p* < .001; ****, *p* < .0001.

The attachment factors promoting CHIKV entry into insect cells are poorly defined. We first evaluated CHIKV infectivity in mosquito cells in the presence of competitors that reduced entry into mammalian cell lines. Mosquitoes do not have a Mxra8 orthologue (Zhang et al., 2018; Zhang et al., 2019), therefore, we did not assess that antibody competition. As anticipated, CHIKV-neutralizing antibodies blocked replication in *Ae. Albopictus* (C6/36) and *Ae. aegypti* (Aag2) cells (Figure 6A). Addition of heparan sulfate did not affect CHIKV infection in C6/36 cells but, interestingly, caused an increase in infection in Aag2 cells (Figure 6B). Neither PS-containing nor PC-only liposomes altered CHIKV infection in mosquito cells (Figures 6C, D), and modulation of viral envelope PS levels did not significantly affect particle infectivity (Figure 6E, Supplementary Figure S4). Together, these data suggest CHIKV infection in mosquito cells does not occur through PSRs or heparan sulfate.

To determine if carbohydrates on the viral particle influence CHIKV infection, we treated CHIKV particles with either PNGase F, an enzyme that cleaves N-linked glycosylations, or heparinase II which cleaves heparin and heparan sulfate. Treatment with PNGase F resulted in approximately half of the E1 protein migrating faster on the SDS-PAGE gel, suggesting removal of some of the N-linked glycans (Supplementary Figure S5A). Heparinase II treatment did not result in an observable shift in E1 migration, but because only the terminal sugar moieties would have been removed it was not predicted to significantly alter gel migration. We observed a slight increase (∼45%) in infection of PNGase-treated particles into C6/36 cells (*p* = 0.052) but it did not significantly alter infectivity in Vero, HAP1, or Aag2 cells (Supplementary Figure S5B). Treatment with heparinase did not affect infection of CHIKV in any of the cell lines (Supplementary Figure S5B).

### 3.6 Mosquito cells are sensitive to sodium azide causing inhibition of virus infection

**FIGURE 7:**
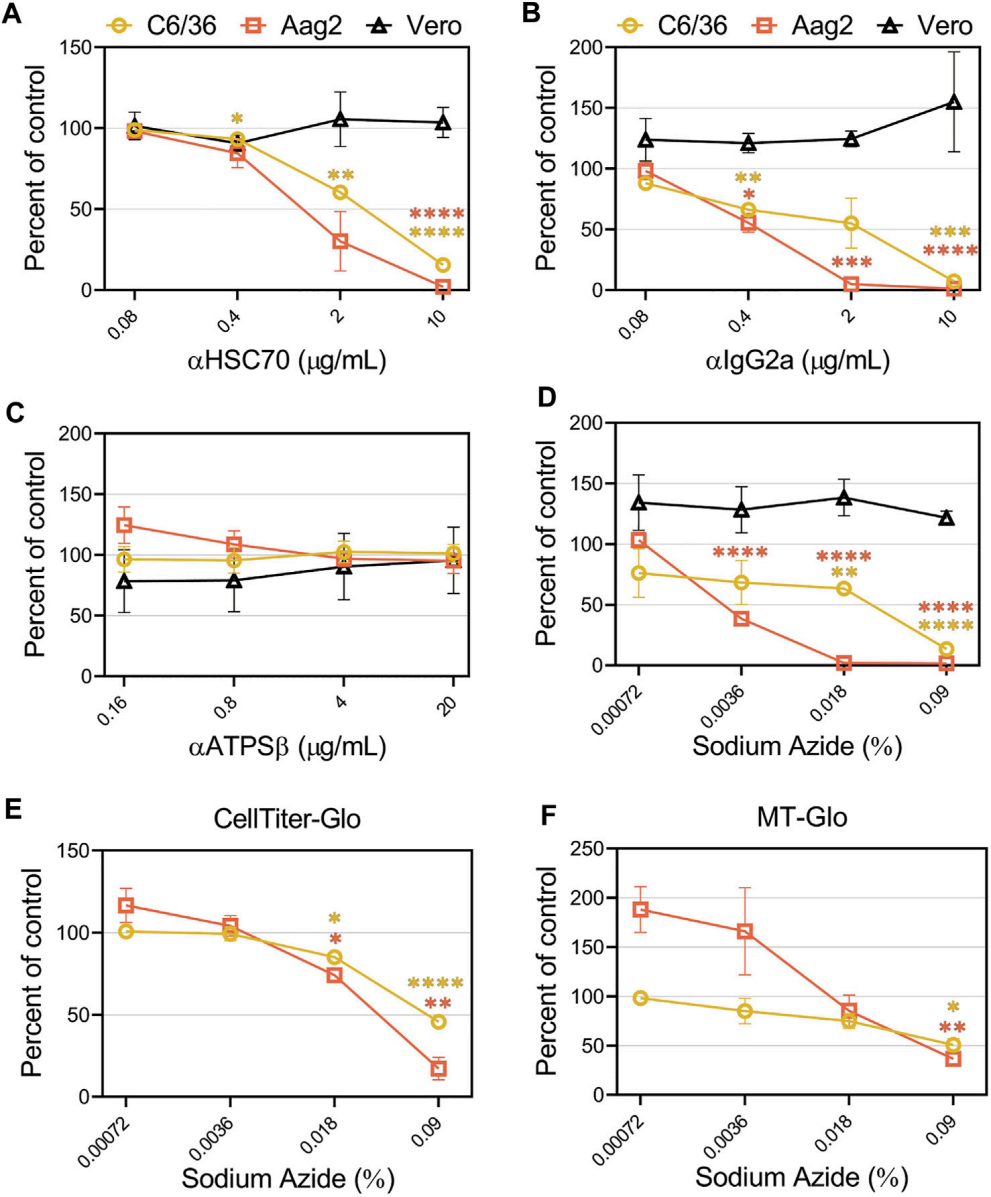
Sodium azide preservatives cause inhibition of CHIKV production in mosquito cells. CHIKV-Nluc stocks were used to assess the ability of **(A)** HSC70, **(B)** IgG2a, **(C)** ATPSβ, or **(D)** sodium azide only to block infections into either C6/36, Aag2, or Vero cells at the indicated concentrations. 24 h following infection the cells were lysed with NanoGlo substrate and lysates were quantified with a GloMax Explorer. Cell viability of infected C6/36 and Aag2 cells was determined after treatment with sodium azide or PBS as control by measuring ATP levels **(E)** or reducing potential of the cells **(F)**. 24 h following infection the cells were lysed with Cell Titer Glo or RealTime-Glo MT, respectively, and quantified with a GloMax Explorer. Data are presented as the mean ± SEM from at least three independent experiments performed in duplicate. Unpaired parametric Student’s t-test with Welch’s correction was performed to determine statistical significance compared to a no-treatment control. *, *p* < .05; ***, *p* < .001; ****, *p* < .0001.

Previous studies suggested CHIKV entry into mosquito cell lines is mediated through heat shock cognate 70 protein (HSC70) and ATP synthase *β* (ATPSβ) (Fongsaran et al., 2014; Ghosh et al., 2017). To confirm these findings, we performed competition assays with antibodies against these proteins in C6/36, Aag2, and Vero cells. The addition of an HSC70 antibody inhibited CHIKV infection in C6/36 and Aag2 cells in a dose-dependent manner but did not alter infection in Vero cells (Figure 7A). However, competition with several negative control antibodies that were either non-specific (IgG2a) or random (XKR8), also decreased CHIKV infection in mosquito cells (Figure 7B and Supplementary Figure S6A). We did not observe inhibition with addition of antibodies targeting ATPSβ (Figure 7C). Upon careful inspection of the composition of the antibodies utilized, we observed a correlation between the amount of sodium azide (NaN_3_) preservative and the degree of CHIKV inhibition (Supplementary Table S1). The highest level of NaN_3_ was present in the IgG2a antibody while the ATPSβ antibody did not contain any NaN_3_. To determine if the inhibition previously observed was due to the level of NaN_3_ present during infection, we performed additional experiments adding either only NaN_3_ or an additional negative control antibody that lacks NaN_3_ (αEBOV) (Figure 7D and Supplementary Figure S6B). Congruent with the αATPSβ lacking NaN_3_, we did not see any inhibition of CHIKV infection in the presence of the EBOV antibody. In contrast, the addition of NaN_3_ alone, at concentrations reflective of its use as an antibody preservative, inhibited CHIKV infection in mosquito cells but not in Vero cells (Figure 7D). We assessed the effect of the same concentrations of sodium azide in the cell viability of C6/36 and Aag2 cells by measuring the ATP levels (Figure 7E) or the reducing potential of the cells (Figure 7F). We observed a decrease in cell viability at high concentrations of NaN_3_. These data suggest that the inhibition previously observed in C6/36 and Aag2 cells was due to the presence of NaN_3_ preservative in commercially available antibodies.

### 3.7 Vero and Aag2 cells display similar entry efficiency of CHIKV

**FIGURE 8:**
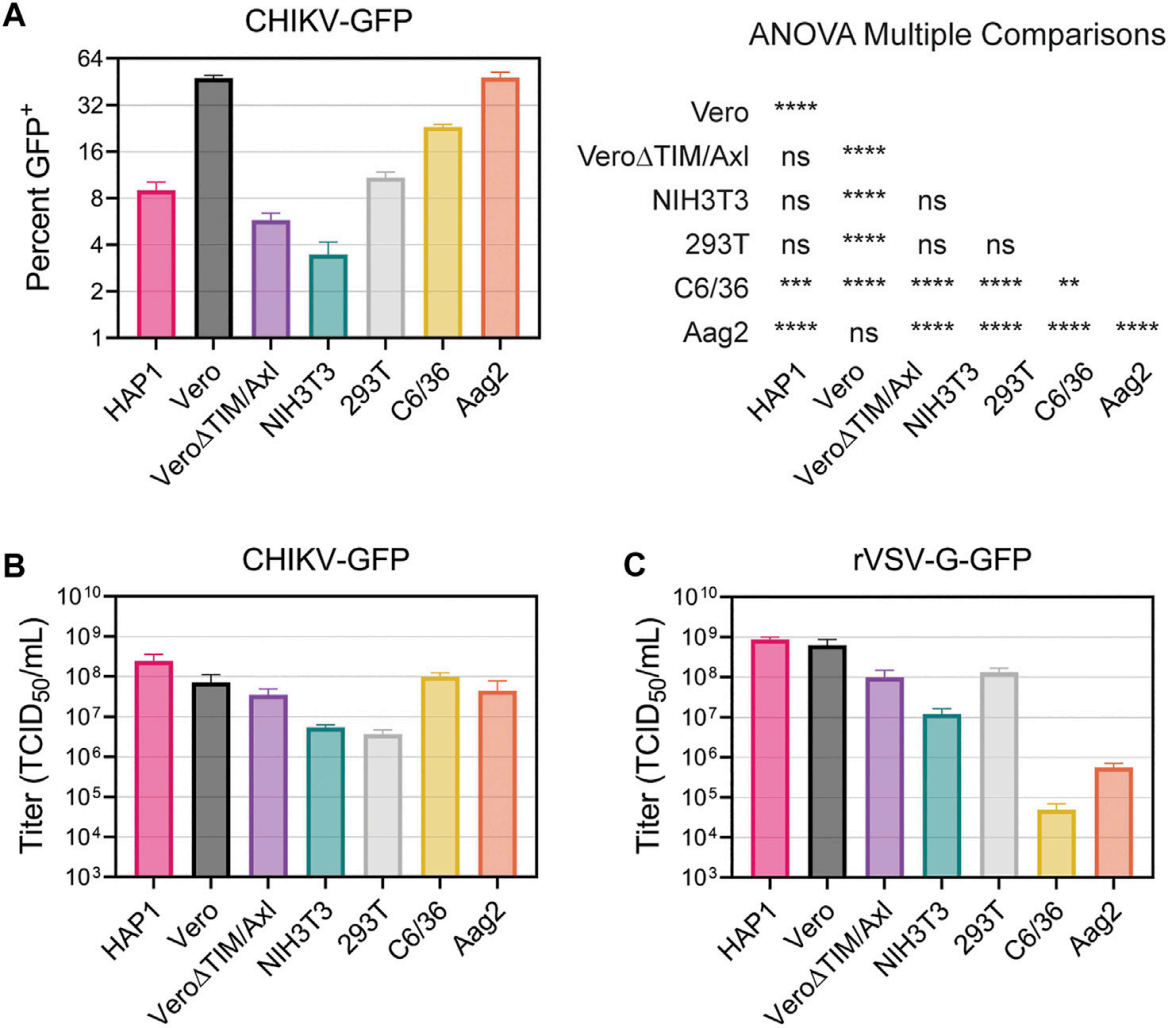
Vero and Aag2 cells display similar entry efficiency of CHIKV. **(A)** CHIKV-GFP entry among all mammalian and mosquito cell lines used was monitored after one round of replication. Percent of GFP^+^ cells was determined through flow cytometry. Statistical significance corresponding to entry efficiency was calculated with a multiple comparisons ANOVA analysis: *, *p* < .05; ***, *p* < .001; ****, *p* < .0001. Viral stocks of **(B)** CHIKV-GFP 181/c25 **(C)** rVSV-G-GFP were titrated in each mammalian and mosquito cells used. Data are presented as the mean ± SEM from three independent experiments. The results from the ANOVA with multiple comparisons can be found in Supplementary Table S2.

Finally, we directly compared the ability of CHIKV virions to enter and spread among all the cell lines evaluated. First, we added CHIKV-GFP to each cell line for 2 hours, removed the inoculum, and prevented subsequent rounds of infection by adding NH_4_Cl to the media. After 18 h, the number of virally infected cells was enumerated in the flow cytometer. Vero and Aag2 cells displayed the greatest number of CHIKV-infected cells, followed by C6/36 cells (Figure 8A). In contrast, we observed significantly lower numbers of infected HAP1, Vero∆TIM/Axl, NIH3T3, and 293T cells (Figure 8A). Next, we serially diluted the viral inoculum and added it to each cell line to determine the relative viral titer. CHIKV-GFP produced similar TCID_50_ values in HAP, Vero, Vero∆TIM/Axl, C6/36, and Aag2 cell lines, while the same stock displayed a 10-fold lower TCID_50_ value when added to NIH3T3 and 293T cells (Figure 8B). This was a CHIKV specific phenotype, given that titers of VSV-G displayed distinct relative titers in 293T and mosquito cells (Figure 8C).

We utilized recombinant VSV particles encoding its native glycoprotein or CHIKV glycoproteins from either the Asian 181/c25 or East-Central-South-African (ECSA) S27 strains to evaluate strain differences in CHIKV titers among cell lines (Supplementary Figures S7A, B). We did not observe any major differences in titer trends between the two CHIKV glycoproteins. Interestingly, while the CHIKV-GFP viral titers were not significantly different when comparing Vero and Vero∆TIM/Axl, both rVSV∆G-181/c25 and rVSV∆G-S27 titers were 10-fold higher in Vero cells than Vero∆TIM/Axl. Further, C6/36 cells were not very permissive to rVSV particles containing its native glycoprotein or the strain-specific glycoprotein from CHIKV (Figure 8C, Supplementary Figures S7A, B).

## 4 Discussion

Efficient viral replication requires many cellular factors, some of which are involved in viral attachment and entry, and others are required for optimal replication to occur. CHIKV displays a wide cell and species tropism (Salvador et al., 2009; Matusali et al., 2019), suggesting it may utilize ubiquitous host factors that are conserved between the mosquito and mammalian hosts. Although several host factors facilitate CHIKV infection, none have been shown to be essential for productive infection. A ubiquitous attachment mechanism among all CHIKV susceptible cell lines may exist, but its identification has remained elusive. Alternatively, an array of studies on CHIKV entry suggests several disparate molecules, including proteins, carbohydrates, and lipids can mediate particle attachment at the virion-cell interface (Fongsaran et al., 2014; Silva et al., 2014; Ghosh et al., 2017; Zhang et al., 2018; Zhang et al., 2019; McAllister et al., 2020; Kirui et al., 2021).

In this study, we compared the entry requirements for CHIKV in several mammalian and mosquito cell lines. Each mammalian cell line we examined varied in which attachment factor contributed more prominently to CHIKV infection. Overall, CHIKV infection proceeded most efficiently in the presence of either: HS in HAP1, PSRs in Vero cells, and Mxra8 in NIH3T3. While the majority of CHIKV entry occurred through different attachment factors, additional less efficient routes enabled entry in each cell line. In some cell lines, such as Vero cells, attachment appears highly efficient as additional factors did not further enhance infection. In contrast, CHIKV infection can be significantly enhanced in other cell lines such as HAP1 and 293T cells. Entry into mosquito cells appears to be independent of these mammalian attachment molecules. Additional proteins (e.g., C-type Lectins and Prohibitin-1) have been suggested to facilitate CHIKV infection (Wintachai et al., 2012; Bucardo et al., 2020), which were not examined in this study. Further, on investigation of previously implicated mosquito cell attachment factors HSC70 and ATPSβ, we discovered that the sodium azide present in many commercial antibodies can block CHIKV inhibition in commonly used mosquito C6/36 and Aag2 cells.

### 4.1 Relative contribution of each attachment factor in mammalian cells

Mxra8 was found to mediate the entry of CHIKV into NIH3T3 cells by a CRISPR-Cas9 screen (Zhang et al., 2018). While the presence of Mxra8 clearly enhances CHIKV infection in some cells, it is not required for infection in many cell lines. CHIKV can infect Mxra8-deficient mice and mosquitoes lacking a Mxra8 orthologue, suggesting other pathways must also be used (Zhang et al., 2018). NIH3T3 cells produce Mxra8 and high levels of HS on their cell surface, but do not produce TIM-1 (Kobayashi et al., 2007; Jemielity et al., 2013; McAllister et al., 2020). CHIKV infection was efficiently blocked with Mxra8 antibodies (Figure 3D), whereas the addition of HS did not significantly alter infection (Figure 3E). This suggests that, in NIH3T3 cells, Mxra8 is more important for entry than HS interactions. NIH3T3 cells were infected relatively poorly compared to the other cell lines (Figures 8A, B). They also displayed lower VSV titers (Figure 8C), suggesting they may be less permissive and prevent viral replication through an innate mechanism.

Like Mxra8, GAG production enhances CHIKV infection (Silva et al., 2014; McAllister et al., 2020). While NIH3T3, Vero, and HAP1 cells endogenously produce GAGs (McAllister et al., 2020), we found that heparan sulfate only competitively inhibited CHIKV infection in HAP1 cells. HAP1 cells also displayed the highest titers (Figure 3A; Figure 8B), but when the virus was added for a short time period, infection was relatively low, similar to NIH3T3 and Vero∆TIM/Axl cells (Figure 8A). CHIKV entry into HAP1 cells was enhanced with exogenous Mxra8, but not TIM-1 (Figure 1A), suggesting Mxra8 can enhance entry during a short infection time above the level provided by the naturally produced GAGs. These differences in infection suggest GAGs mediate CHIKV attachment but are inefficient and require additional time to capture viral particles effectively. Similar results are seen with other GAG-utilizing viruses. For example, herpes simplex virus 1 interacts with HS, mediating close contact with the cell and facilitating binding to additional receptors in the cell surface (Shieh et al., 1992; Montgomery et al., 1996).

The attenuated CHIKV strain used in this study (181/c25) displays increased GAG dependence compared to circulating pathogenic strains based on interactions with residue 82 on E2 (Ashbrook et al., 2014; Silva et al., 2014; Hawman et al., 2016; Sahoo and Chowdary, 2019; McAllister et al., 2020). The degree of GAG dependence appears to be strain specific (McAllister et al., 2020). Given that we observed PS-dependent entry in Vero cells using a CHIKV strain with strong GAG affinity suggests that endemic strains may either 1) be more reliant on alternative attachment factors and/or 2) be less infectious in the same context. A recent study found that exogenous production of TIM-1 in 293T increased CHIKV infection with East-Central-South-African (ECSA), West African (WA), and Asian (181/c25) strains (Kirui et al., 2021). Similarly, we did not observe any major differences between the CHIKV 181/c25 and S27 envelopes when titrating the viral stocks in the different cell lines (Supplementary Figures S7A, B) suggesting that the efficiency of each entry pathway is more dependent on the host cell than the strain of CHIKV.

While Vero cells naturally produce PSRs (i.e., TIM-1 and Axl) (Brouillette et al., 2018) and Mxra8, CHIKV entry is highly dependent on TIM-1 (Figure 3F) and was unaffected by the addition of Mxra8 antibody (Figure 3D). CHIKV produced the highest level of GFP^+^ cells when entering Vero cells and removal of the PSRs significantly decreased entry (Figure 8A). Additionally, virion particle PS levels correlated with specific infectivity when infecting Vero cells (Figure 5, Supplementary Figure S3) and PS containing liposomes blocked infection. These data suggest TIM-1 and apoptotic mimicry are important for efficient entry into Vero cells. The addition of other attachment factors did not enhance entry into Vero cells (Figure 2A), suggesting Vero cells are efficient at capturing PS-containing cargo. Removal of TIM-1 and Axl from Vero cells renders them less susceptible to CHIKV infection but given enough time, the virus can enter (Figure 4B). Therefore, in the absence of PSRs, the molecular components facilitating virion attachment and entry in Vero cells are sufficient but inefficient.

### 4.2 Viral entry mechanisms into mosquito cells

Transmission of CHIKV to a mosquito vector can occur when a susceptible mosquito ingests blood from a viremic mammalian host. The literature exploring the host factors used by CHIKV to enter mosquito cells is limited. We evaluated the role of the previously identified binding partners during CHIKV infection of *Aedes* C6/36 and Aag2 cells. Neither HS nor PS inhibited CHIKV infection in mosquito cells and modulation of envelope PS did not affect the infectivity of the virus (Figure 6). Given the cell line specific effects observed across mammalian cell lines, it should be noted that Aag2 cells are derived from larval homogenates and are not clonal cells.

Previous studies aiming to identify a receptor for CHIKV in mosquito cells suggest HSC70 (Ghosh et al., 2017) and ATPSβ (Fongsaran et al., 2014) may be important entry factors. HSC70 is a chaperone protein that has been associated with many cellular processes including protein translocation, folding, and stabilization (Bukau and Horwich, 1998). ATPSβ is a mitochondrial protein that drives ATP synthesis (Leyva et al., 2003). The proposed role of these proteins in CHIKV infection was previously demonstrated through antibody-mediated inhibition assays. We were able to obtain the same HSC70 antibody employed in the prior study. Unfortunately, the ATPSβ antibody used previously was no longer available, therefore we purchased another polyclonal antibody that was made using a similar immunogen. While we observed dose-dependent inhibition of CHIKV infection in the presence of the HSC70 antibody, no effect was observed with the ATPSβ antibody (Figures 7A, C). Several control antibodies also displayed inhibition of CHIKV infection (Figure 7B and Supplementary Figure S6A). Upon careful observation, we noted the inhibition correlated with the level of a commonly added antibody preservative, sodium azide. The ATPSβ antibody used in the previous paper contained NaN_3_ as well, which may have produced the inhibitory results. NaN_3_ is a highly toxic chemical that prevents proper phosphorylation and cytochrome oxidation. NaN_3_ inhibits mitochondrial respiration in C6/36 cells at much lower concentrations than in mammalian cells (Santana-Román et al., 2021) and efficiently blocked CHIKV replication in mosquito cells (Figure 7). Future studies should carefully consider the composition of reagents when evaluating the role of proteins in mosquito cells. The previous work also used RNAi against ATPSβ or inhibitors against HSC70 which both reduced CHIKV levels (Fongsaran et al., 2014; Ghosh et al., 2017). Reducing the levels/activities of either ATPSβ or HSC70 would be expected to decrease cellular metabolism or protein folding and inadvertently decrease CHIKV replication. Additional studies should follow up the roles of these proteins in CHIKV infection.

The entry factors responsible for CHIKV infection in mosquito cells remain unknown. However, many differences exist between mammalian and mosquito cells that could result in the expression of distinct cellular attachment factors important for CHIKV. For example, differences in protein post-translation modifications between mammalian and invertebrate cells can contribute to differences in exposed cellular surface glycans (e.g., N-glycosylation) available for attachment. Trimmed glycans produced in the endoplasmic reticulum of vertebrate cells travel to the Golgi where they encounter acetylglucosaminyl-, galactosyl-, and sialyl-transferases that mediate branching events to produce hybrid and complex glycans (Turco and Robbins, 1978). Mosquito cells do not produce these transferases, creating mostly high-mannose or paucimannose glycans (Butters et al., 1981; Hsieh and Robbins, 1984). We observed a slight increase in infection of C6/36 cells with PNGase-treated CHIKV virions (Supplementary Figure S5). This suggests that the removal of highly branched N-linked glycans derived from the mammalian host cells might enhance interactions with CHIKV mosquito receptors.

In addition, the plasma membrane of insect cells has a distinct lipid profile from that of mammalian cells (Shiomi et al., 2021). Vertebrate cells synthesize cholesterol, one of the main mediators of membrane fluidity (Yeagle, 1985). The inability of insect cells to *de novo* synthesize cholesterol leads to modulation of the production of other phospholipids (Dawaliby et al., 2016; O’Neal et al., 2020). Insect cells display a two-fold increase in the production of phosphatidylethanolamine (PE) compare to mammalian cells (Dawaliby et al., 2016). Not only is the amount of this aminophospholipid different, but its distribution in the plasma membrane of insect cells is also altered (Shiomi et al., 2021). The plasma membrane of mammalian cells exhibits a characteristic phospholipid asymmetry where PE and phosphatidylserine (PS) are maintained in the inner leaflet (Murate et al., 2015). These phospholipids are exposed after signaling events that trigger the activation of scramblases. Previous studies have shown that the constitutive activation of XKR scramblases exhibited by arthropod cells leads to a symmetrical distribution of phospholipids in the plasma membrane (Shiomi et al., 2021). Thereby increasing the amount of PE consistently exposed in the exoplasmic leaflet, relative to mammalian cells. Studies evaluating the presence of phospholipid-binding receptors in mosquito cells are limited. Although we did not observe the role of PS-binding receptors in CHIKV entry into mosquito cells (Figure 6), future studies should evaluate other lipid-binding proteins that may mediate CHIKV infection. TIM-1 orthologs have not been found in mosquito cells, but *drosophila* encodes PSR orthologs, and apoptotic cell clearance *via* phosphatidylserine exposure is conserved (van den Eijnde et al., 1998).

### 4.3 The potential role of apoptotic mimicry during natural infection

In humans, CHIKV infection is initiated by virion deposition into the skin dermis during the bite of an infectious female mosquito. Fibroblasts, keratinocytes, and resident macrophages support initial CHIKV infection (Bernard et al., 2015; Zhang et al., 2018). Fibroblasts are permissive for CHIKV and the infection appears to be predominately Mxra8-dependent (Zhang et al., 2018). Keratinocytes present in the basal layer of the skin *epidermis* produce both TIM-1 and Axl (Bauer et al., 2012; Dejarnac et al., 2018) and are susceptible to CHIKV infection (Zhang et al., 2018). A recent study demonstrated that the keratinocyte cell line, HaCat, produced low levels of Axl along with undetectable levels of TIM-1 and that the addition of TIM-1 increased CHIKV susceptibility and permissivity (Kirui et al., 2021). Thus, keratinocytes may have a larger role in CHIKV infection establishment *in vivo* than previously thought. Macrophages also display PSRs, conferring phagocytic properties of apoptotic body clearance (Freeman et al., 2010; Wang et al., 2013; Pietkiewicz et al., 2015). PS-rich virions from either infected fibroblasts, keratinocytes, or mosquito inoculation may serve as an ideal target to attach to PSRs on resident macrophages. While macrophage infection *via* apoptotic mimicry could facilitate CHIKV dissemination *in vivo*, macrophages often are poor producers of CHIKV virus *in vitro* (Sourisseau et al., 2007).

Understanding the entry requirements for attachment broadens our understanding of the molecular basis for the wide tissue tropism of CHIKV. Several entry pathways may exist through attachment factor binding in isolation or the involvement of cooperative interactions in a concerted binding-internalization process. However, the delineation of CHIKV virus-cell protein interactions leading to particle internalization across multiple cell lines is currently lacking. The complexity of CHIKV entry warrants future screens to adopt creative approaches to identify the host factors necessary for CHIKV infection among cell lines. Viral establishment, dissemination, and the cross-species transmission of CHIKV between mammalian and mosquito hosts are likely influenced by the assortment of cellular attachment factors across cells.

## Data Availability

The original contributions presented in the study are included in the article/Supplementary Material, further inquiries can be directed to the corresponding author.
