# Supplementary material for: Chikungunya virus entry and infectivity is primarily facilitated through cell line dependent attachment factors in mammalian and mosquito cells
Source: Front Cell Dev Biol. 2023 Jan 20;11:1085913. doi: 10.3389/fcell.2023.1085913 (PMC9895848; doi:10.3389/fcell.2023.1085913)
Supplement: Supplementary file 1 [file DataSheet1.PDF]

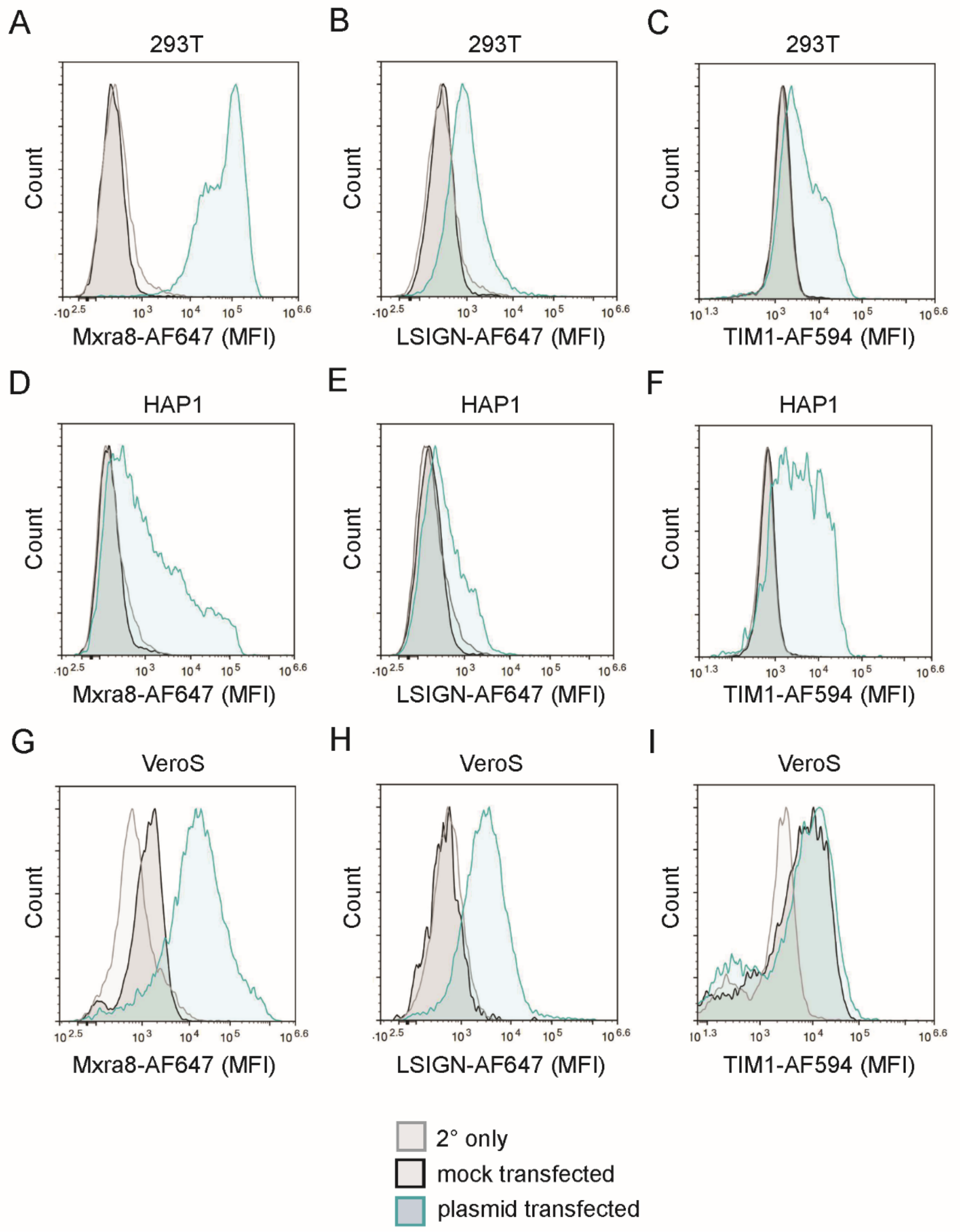


**Supplemental Figure 1. Endogenous and exogenous expression of Mxra8, L-SIGN, and TIM-1 in 29eT, HAP1 and VeroS cells.** Surface presentation of known CHIKV attachment factors (Mxra8, L-SIGN, or TIM-1) was assessed before and after transfection via flow cytometry in **(A-C)** 293T, **(D-F)** HAP1, or **(G-I)** VeroS.

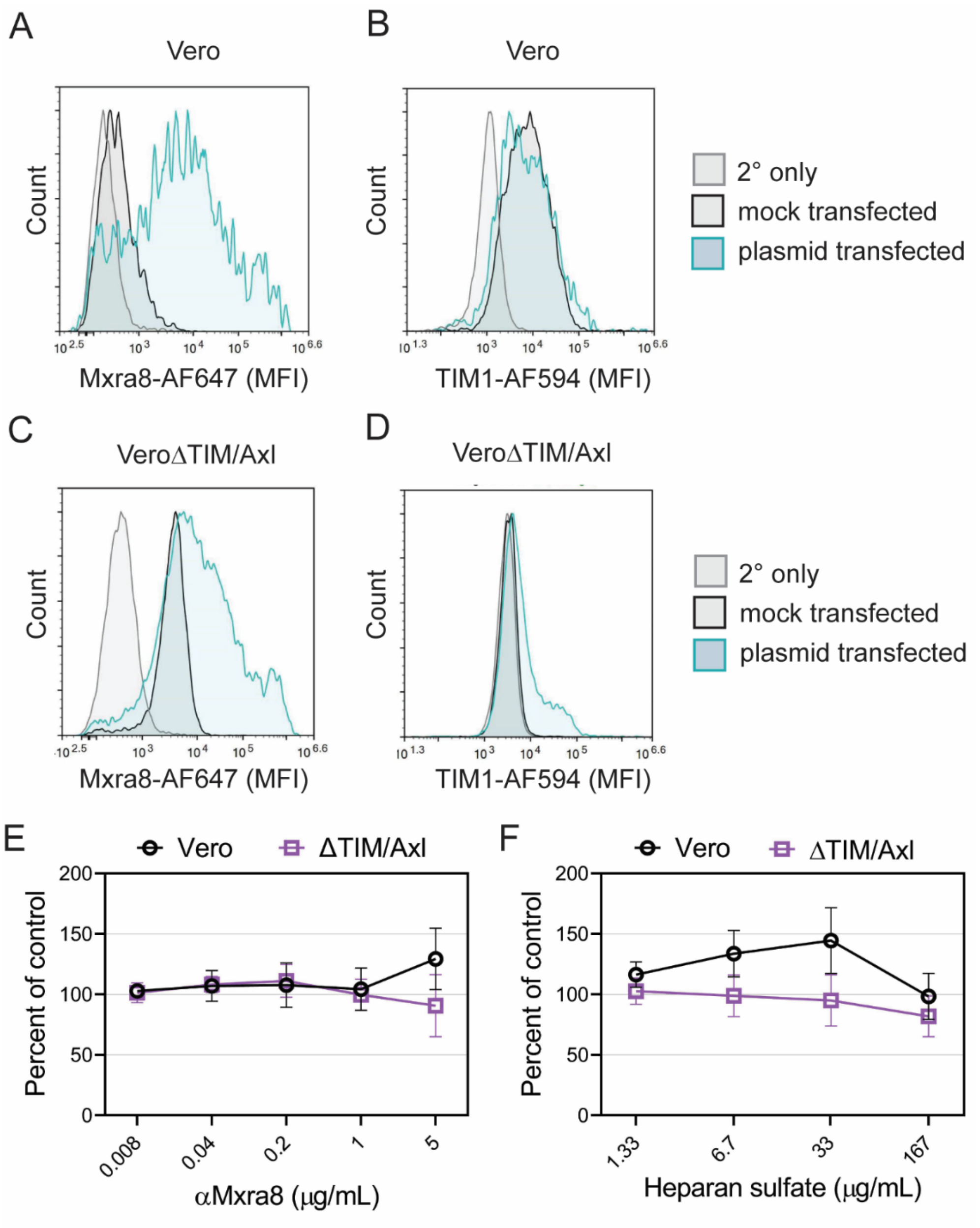


**Supplemental Figure 2. CHIKV entry into VeroΔTIM/Axl is not inhibited by Mxra8 antibodies or HS.** Surface staining of attachment factors, Mxra8 and TIM-1, in Vero **(A-B)** and VeroΔTIM/Axl **(C-D)** cells before and after transfection using flow cytometry. The ability of **(E)** Mxra8 antibody and **(F)** heparan sulfate to block CHIKV infection into VeroΔTIM/Axl cells was assessed at the indicated concentrations. Vero inhibition assay data shown in Figure 3 are presented again for comparison as VeroΔTIM/Axl cells were included as a treatment group during the experiment. Data are presented as the mean ± SEM from three independent experiments performed in triplicate. Unpaired parametric Student's T-test was performed to determine statistical significance **(E-F)** with unequal variance (Welch's correction) compared to a no-treatment control. ***, $p < 0.001$.

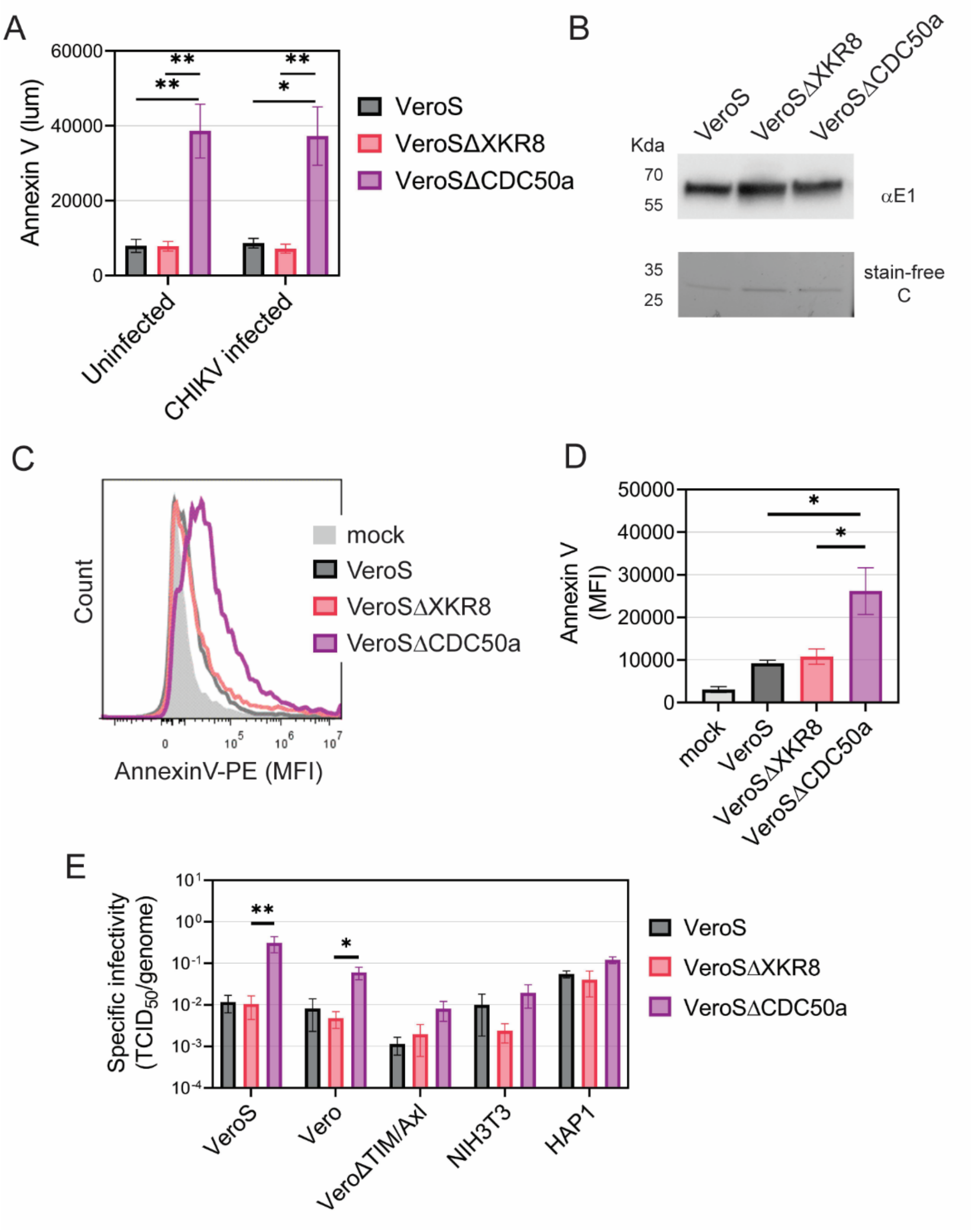


**Supplemental Figure 3. Altering PS levels in CHIKV virion envelope produced in Vero knockout cells recapitulates phenotypes observed with HAP1 cell lines. (A)** VeroS cell lines were monitored for Annexin V binding (luminescence) at 24 hrs using a GloMax Explorer microplate reader. Parental, ΔXKR8, and ΔCDC50a cells were either untreated or infected with CHIKV (strain 181/c25, MOI 1.0). At least three independent replicates were conducted with bars representing the mean and error (±SEM). To quantify levels of externalized PS on the CHIKV viral particle, CHIKV was propagated through the VeroS knockout cell lines. **(B)** Viral inputs were immunoblotted with a CHIKV antibody and assessed for purity using a stain-free gel. **(C-D)** Annexin V conjugated to PE was used to stain normalized amounts of virus-bound beads and quantified via FACs analysis. A bead-only control (mock) was used to establish a baseline signal. MFI values from three independent trials were normalized to parental values (VeroS) with the mean and ±SEM displayed. An unpaired parametric T-test with Welch's correction was used test for statistical significance. **(E)** The ratio of $TCID_{50}$ to particle (genome copy equivalents) for each sample was used to assess the infectivity of particles produced from VeroS cell lines on a panel of mammalian cells (Vero, VeroΔTIM/Axl, NIH3T3, and HAP1). Infectivity values were natural log (ln) transformed prior to performing an unpaired parametric student T-test. At least three independent replicates were conducted with bars representing the mean and error (±SEM).

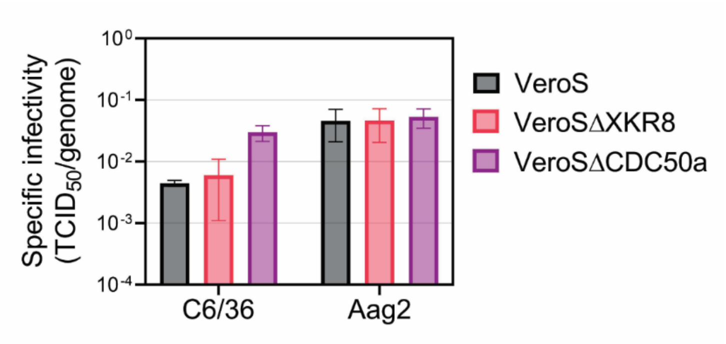


**Supplemental Figure 4. Modulation of viral envelope PS levels does not alter the infectivity of CHIKV in mosquito cells.** The ratio of $TCID_{50}$ to particle (genome copy equivalents) for each sample was used to assess the infectivity of particles produced from VeroS cell lines on a panel of mosquito cells (C6/36 and Aag2). Infectivity values were natural log (ln) transformed prior to performing an unpaired parametric student T-test. At least three independent replicates were conducted with bars representing the mean and error (±SEM).

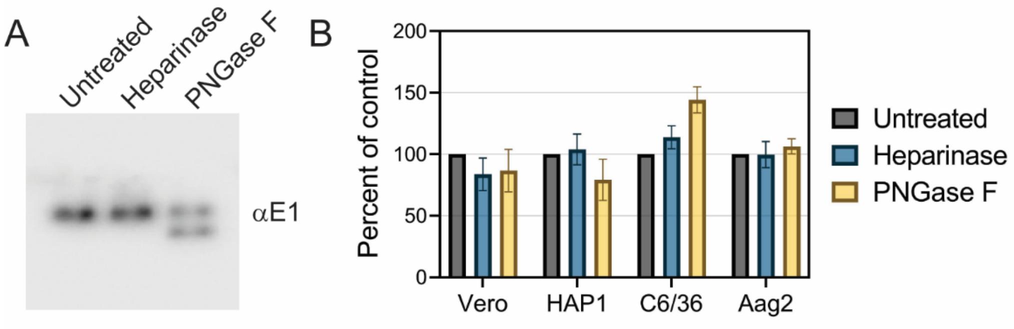


**Supplemental Figure 5. CHIKV infection of mosquito cells is not affected by the loss of N-linked glycans or glycosaminoglycans.** CHIKV-Nluc virions were treated with either PNGase F, Heparinase, or untreated. Treated and untreated virions were **(A)** immunoblotted with an antibody against CHIKV E1 glycoprotein or **(B)** used for infection of Vero, HAP1, C6/36, and Aag2. At 24hpi cells were lysed with NanoGlo substrate and lysates were quantified with a GloMax Explorer. Data are presented as the mean ± SEM from three independent experiments performed in triplicate. Unpaired parametric Student's T-test with unequal variance (Welch's correction) was performed to determine statistical significance compared to a no-treatment control.

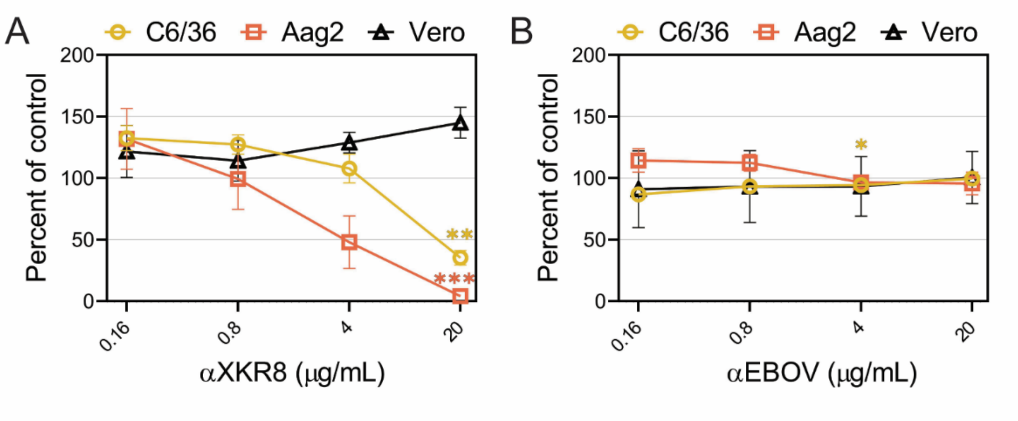


**Supplemental Figure 6. Sodium azide inhibits CHIKV infection in mosquito cell lines.** CHIKV-Nluc stocks were used to assess the ability of **(A)** XKR8 or **(B)** EBOV antibodies to block infections into either C6/36, Aag2, or Vero cells at the indicated concentrations. Twenty-four hours following infection the cells were lysed with NanoGlo substrate and lysates were quantified with a GloMax Explorer. Data are presented as the mean ± SEM from at least three independent experiments performed in duplicate. Unpaired parametric Student's T-test with Welch's correction was performed to determine statistical significance compared to a no-treatment control.*, $p < 0.05$; ***, $p < 0.001$; ****, $p < 0.0001$.

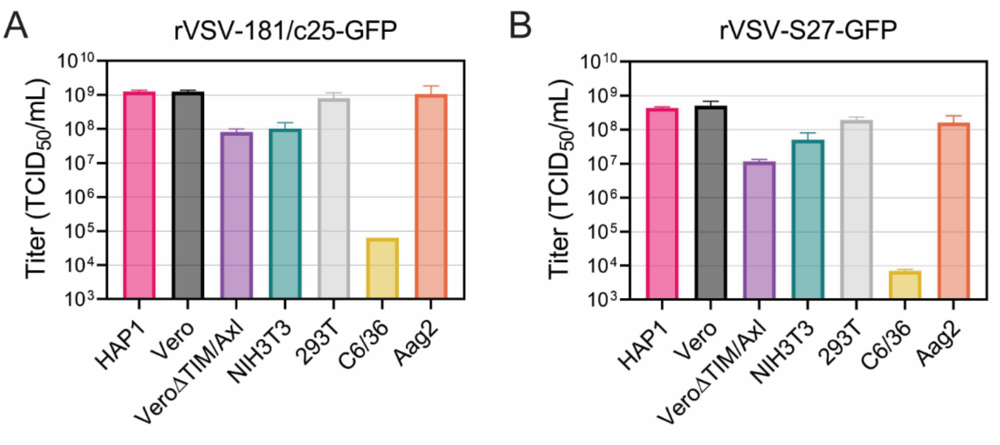


**Supplemental Figure 7. Titers of rVSV particles containing Asian and East-Central-South-African CHIKV envelopes are similar across cell lines.** Viral stocks of **(A)** rVSV-181/c25-GFP, **(B)** rVSV-S27-GFP, were titrated in mammalian and mosquito cells used. Data are presented as the mean ± SEM from three independent experiments. The results from the ANOVA with multiple comparisons can be found in Supplemental table 2.

**Supplemental Table 1. Percentage of sodium azide present in antibodies**

| Antibody | Catalog Number | Sodium Azide (%) |
|---|---|---|
| HSC70 monoclonal | Invitrogen, cat. MA3-014 | 0.05 |
| Mouse IgG2a K Isotype control eBM2a | eBioscience Inc., cat. 14-472481 | 0.09 |
| XKR8 antibody | ThermoFisher, cat. PA5-65799 | 0.02 |
| ATP5B pAb | Abnova, cat. H00000506-D01P | 0 |
| human EBOV monoclonal KZ52 | IBT, cat. 0260-001 | 0 |
| ATP5B pAb (48) | Santa Cruz Biotech, cat. sc-16690 | <0.1 |
| CHIKV polyclonal | IBT, cat. 04-008 | 0 |

**Supplemental Table 2. ANOVA with multiple comparisons for Figure 8B and C and Supplemental Figure 7A and B**

| CHIKV-GFP titers | | | | rVSV-G-GFP | | |
|---|---|---|---|---|---|---|
| **Tukey's multiple comparisons test** | **Summary** | **Adjusted P Value** | | **Tukey's multiple comparisons test** | **Summary** | **Adjusted P Value** |
| HAP1 vs. Vero | ns | 0.5012 | | HAP1 vs. Vero | ns | 0.9746 |
| HAP1 vs. VeroΔPSR | ns | 0.1948 | | HAP1 vs. VeroΔPSR | ** | 0.0039 |
| HAP1 vs. NIH3T3 | ** | 0.0021 | | HAP1 vs. NIH3T3 | **** | <0.0001 |
| HAP1 vs. 293T | *** | 0.0006 | | HAP1 vs. 293T | * | 0.0249 |
| HAP1 vs. C6/36 | ns | 0.9531 | | HAP1 vs. C6/36 | **** | <0.0001 |
| HAP1 vs. Aag2 | ns | 0.0976 | | HAP1 vs. Aag2 | **** | <0.0001 |
| Vero vs. VeroΔPSR | ns | 0.9966 | | Vero vs. VeroΔPSR | * | 0.0249 |
| Vero vs. NIH3T3 | ns | 0.0975 | | Vero vs. NIH3T3 | **** | <0.0001 |
| Vero vs. 293T | * | 0.0278 | | Vero vs. 293T | ns | 0.1441 |
| Vero vs. C6/36 | ns | 0.9768 | | Vero vs. C6/36 | **** | <0.0001 |
| Vero vs. Aag2 | ns | 0.9523 | | Vero vs. Aag2 | **** | <0.0001 |
| VeroΔPSR vs. NIH3T3 | ns | 0.294 | | VeroΔPSR vs. NIH3T3 | * | 0.017 |
| VeroΔPSR vs. 293T | ns | 0.0976 | | VeroΔPSR vs. 293T | ns | 0.9746 |
| VeroΔPSR vs. C6/36 | ns | 0.7359 | | VeroΔPSR vs. C6/36 | **** | <0.0001 |
| VeroΔPSR vs. Aag2 | ns | 0.9999 | | VeroΔPSR vs. Aag2 | **** | <0.0001 |
| NIH3T3 vs. 293T | ns | 0.9967 | | NIH3T3 vs. 293T | ** | 0.0026 |
| NIH3T3 vs. C6/36 | * | 0.0166 | | NIH3T3 vs. C6/36 | **** | <0.0001 |
| NIH3T3 vs. Aag2 | ns | 0.5005 | | NIH3T3 vs. Aag2 | *** | 0.0004 |
| 293T vs. C6/36 | ** | 0.0045 | | 293T vs. C6/36 | **** | <0.0001 |
| 293T vs. Aag2 | ns | 0.1947 | | 293T vs. Aag2 | **** | <0.0001 |
| C6/36 vs. Aag2 | ns | 0.499 | | C6/36 vs. Aag2 | ** | 0.0018 |
| | | | | | | |
| **rVSVΔG-181/c25-GFP** | | | | **rVSVΔG-S27-GFP** | | |
| **Tukey's multiple comparisons test** | **Summary** | **Adjusted P Value** | | **Tukey's multiple comparisons test** | **Summary** | **Adjusted P Value** |
| HAP1 vs. Vero | ns | >0.9999 | | HAP1 vs. Vero | ns | >0.9999 |
| HAP1 vs. VeroΔPSR | ** | 0.0064 | | HAP1 vs. VeroΔPSR | **** | <0.0001 |
| HAP1 vs. NIH3T3 | ** | 0.0064 | | HAP1 vs. NIH3T3 | ** | 0.003 |
| HAP1 vs. 293T | ns | 0.9495 | | HAP1 vs. 293T | ns | 0.6863 |
| HAP1 vs. C6/36 | **** | <0.0001 | | HAP1 vs. C6/36 | **** | <0.0001 |
| HAP1 vs. Aag2 | ns | 0.8428 | | HAP1 vs. Aag2 | ns | 0.2087 |
| Vero vs. VeroΔPSR | ** | 0.0064 | | Vero vs. VeroΔPSR | **** | <0.0001 |
| Vero vs. NIH3T3 | ** | 0.0064 | | Vero vs. NIH3T3 | ** | 0.003 |
| Vero vs. 293T | ns | 0.95 | | Vero vs. 293T | ns | 0.6856 |
| Vero vs. C6/36 | **** | <0.0001 | | Vero vs. C6/36 | **** | <0.0001 |
| Vero vs. Aag2 | ns | 0.8439 | | Vero vs. Aag2 | ns | 0.2083 |
| VeroΔPSR vs. NIH3T3 | ns | >0.9999 | | VeroΔPSR vs. NIH3T3 | ns | 0.3642 |
| VeroΔPSR vs. 293T | ns | 0.0523 | | VeroΔPSR vs. 293T | ** | 0.001 |
| VeroΔPSR vs. C6/36 | **** | <0.0001 | | VeroΔPSR vs. C6/36 | **** | <0.0001 |
| VeroΔPSR vs. Aag2 | ns | 0.0926 | | VeroΔPSR vs. Aag2 | ** | 0.0063 |
| NIH3T3 vs. 293T | ns | 0.0526 | | NIH3T3 vs. 293T | ns | 0.0791 |
| NIH3T3 vs. C6/36 | **** | <0.0001 | | NIH3T3 vs. C6/36 | **** | <0.0001 |
| NIH3T3 vs. Aag2 | ns | 0.093 | | NIH3T3 vs. Aag2 | ns | 0.3657 |
| 293T vs. C6/36 | **** | <0.0001 | | 293T vs. C6/36 | **** | <0.0001 |
| 293T vs. Aag2 | ns | >0.9999 | | 293T vs. Aag2 | ns | 0.9761 |
| C6/36 vs. Aag2 | **** | <0.0001 | | C6/36 vs. Aag2 | **** | <0.0001 |
